# Stabilization of Zwitterionic Versus Canonical Glycine by DMSO Molecules

**DOI:** 10.3390/ph18081168

**Published:** 2025-08-06

**Authors:** Verónica Martín, Alejandro Colón, Carmen Barrientos, Iker León

**Affiliations:** 1 Departamento de Química Física y Química Inorgánica, Universidad de Valladolid, 47011 Valladolid, Spain; veronica.martin.hernandez@uva.es (V.M.); alejandro.colon@estudiantes.uva.es (A.C.); 2 Grupo de Espectrocopía Molecular (GEM), Edificio Quifima, Laboratorios de Espectroscopia y Bioespectroscopia, Unidad Asociada CSIC, Parque Científico UVa, Universidad de Valladolid, 47011 Valladolid, Spain

**Keywords:** glycine, zwitterion, DMSO, computational chemistry, DFT, non-covalent interactions, solvent effects, conformational analysis, QTAIM, biomolecular stabilization

## Abstract

**Background/Objectives**: Understanding the stabilization mechanisms of amino acid conformations in different solvent environments is crucial for elucidating biomolecular interactions and crystallization processes. This study presents a comprehensive computational investigation of glycine, the simplest amino acid, in both its canonical and zwitterionic forms when interacting with dimethyl sulfoxide (DMSO) molecules. **Methods**: Using density functional theory (DFT) calculations at the B3LYP/6-311++G(d,p) level with empirical dispersion corrections, we examined the conformational landscape of glycine–DMSO clusters with one and two DMSO molecules, as well as implicit solvent calculations, and compared them with analogous water clusters. **Results**: Our results demonstrate that while a single water molecule is insufficient to stabilize the zwitterionic form of glycine, one DMSO molecule successfully stabilizes this form through specific interactions between the S=O and the methyl groups of DMSO and the NH_3_^+^ and the oxoanion group of zwitterionic glycine, respectively. Topological analysis of the electron density using QTAIM and NCI methods reveals the nature of these interactions. When comparing the relative stability between canonical and zwitterionic forms, we found that two DMSO molecules significantly reduce the energy gap to approximately 12 kJ mol^−1^, suggesting that increasing DMSO coordination could potentially invert this stability. Implicit solvent calculations indicate that in pure DMSO medium, the zwitterionic form becomes more stable below 150 K, while remaining less stable at room temperature, contrasting with aqueous environments where the zwitterionic form predominates. **Conclusions**: These findings provide valuable insights into DMSO’s unique role in biomolecular stabilization and have implications for protein crystallization protocols where DMSO is commonly used as a co-solvent.

## 1. Introduction

Amino acids are fundamental organic compounds for life, as they constitute the basic units of proteins, playing essential roles in numerous biochemical processes. Their behavior in different solvation environments determines critical aspects such as protein folding, conformational stability, and chemical reactivity [1,2]. Therefore, a detailed understanding of how the solvation environment affects the properties of amino acids is crucial for various fields, from structural biochemistry to drug design and materials science. Previous studies have demonstrated that the presence of solvent molecules can significantly alter the physicochemical properties of amino acids [3,4,5]. In particular, the transition between neutral and zwitterionic forms represents a fundamental phenomenon that reflects the sensitivity of these molecules to their microenvironment. This transition affects not only the net charge of the amino acid but also its hydrogen bonding properties, dipole moment, and spectroscopic behavior.

With the above in mind, the stabilization of amino acids in different solvent environments has represented a fundamental area in understanding molecular interactions in biological and chemical systems. Glycine, structurally the simplest amino acid, constitutes an ideal model for studying these phenomena due to its ability to exist in different forms, including the zwitterionic form that predominates in aqueous environments. The conformational behavior of amino acids is strongly influenced by their environment. While the neutral form is energetically favorable in the gas phase, the zwitterionic form prevails in aqueous solution due to specific interactions with water molecules. This conformational equilibrium has been extensively studied in aqueous media for most amino acids [6,7,8,9], establishing that explicit solvation plays a crucial role in stabilizing the zwitterionic form.

Dimethyl sulfoxide (DMSO), on the other hand, also represents a solvent of great interest in chemistry and biology due to its unique properties as a polar aprotic solvent [10]. Its high dipole moment (3.96 D), combined with its ability to act as a hydrogen bond acceptor but not as a donor, gives it distinctive characteristics compared to other solvents. DMSO has a relatively high dielectric constant (ε = 46.7 at 25 °C), which facilitates the solvation of ionic and polar compounds [11]. In the context of biomolecule crystallization, DMSO plays a crucial role as a precipitation agent [12,13] and as a medium to control nucleation and crystal growth. Solvents used in biological applications are often chosen through trial and error, and DMSO has proven to be one of the most versatile due to its ability to penetrate and diffuse easily through the biological membranes of bacteria and cells. Additionally, its ability to interact with polar groups of proteins and amino acids, combined with its low volatility and thermal stability, makes it a valuable component in crystallization protocols. Furthermore, DMSO can influence the conformation of peptides and proteins, potentially altering secondary and tertiary structures, which has significant implications for X-ray crystallography and other structural techniques. At low concentrations, DMSO stabilizes the folded state of proteins through preferential hydration [14]. However, as the DMSO concentration increases, its interaction shifts from preferential hydration to preferential binding with proteins, resulting in structural changes and unfolding [12,13] so it can denature proteins.

Advances in computational chemistry have provided powerful tools to investigate solute–solvent interactions at the molecular level. Methods based on quantum mechanics, such as density functional theory (DFT), allow precise modeling of the electronic effects that govern interactions between amino acids and solvent molecules [15,16]. These methods offer significant advantages for exploring complex systems where specific interactions, such as hydrogen bonds and electrostatic forces, play a determining role. Solvent modeling can be approached through two main approaches: implicit and explicit models. Implicit models, such as the polarizable continuum model (PCM) [17] or the solvation model based on electron density (SMD) [18], treat the solvent as a continuous medium characterized by its dielectric constant. Although computationally efficient, these models do not capture specific solute–solvent interactions. On the other hand, explicit models incorporate discrete solvent molecules, allowing a more realistic representation of intermolecular interactions, albeit with a higher computational cost. The combination of both approaches, known as a hybrid solution, represents a promising strategy to balance accuracy and computational efficiency. This approach is particularly valuable for studying systems such as glycine in DMSO, where specific interactions with a limited number of solvent molecules can determine conformational stability.

The scientific literature extensively documents the stabilization of the zwitterionic form of amino acids in aqueous media [19,20,21]. Pioneering works indicated that a single water molecule is sufficient to stabilize the glycine zwitterion in the gas phase [22,23,24]. However, other studies demonstrated that at least two water molecules are required to stabilize the glycine zwitterion in the gas phase [25]. In contrast, studies on zwitterion stabilization in DMSO are comparatively scarce. Experimental investigations using IR and NMR spectroscopy have provided evidence for the existence of zwitterionic forms of some amino acids in DMSO, but the underlying molecular mechanisms and specific solvation requirements remain insufficiently understood. This gap in knowledge underscores the importance of detailed computational studies that can elucidate the factors governing the conformational stability of amino acids in this solvent.

The direct comparison between solvation in water and in DMSO is particularly interesting due to the fundamental differences in the properties of these solvents. While water can act as both a donor and acceptor of hydrogen bonds, it is usually thought that DMSO can only function as an acceptor and that weak methyl hydrogen to electronegative intermolecular contacts probably do not constitute hydrogen bonds [26,27,28,29,30]. This difference has significant implications for the hydrogen bond network that can form around the amino acid and, therefore, for the relative stabilization of different conformational forms. The crystallization of proteins and peptides constitutes a critical step in structural determination by X-ray crystallography, a fundamental technique in structural biology. The crystallization process depends sensitively on solvent conditions, including composition, concentration, and physicochemical properties of the medium. DMSO, as a frequent component in crystallization cocktails, can significantly influence this process. Understanding how DMSO interacts with amino acids at the molecular level provides valuable information for optimizing crystallization protocols. DMSO’s ability to stabilize certain amino acid conformations can affect the formation of intermolecular contacts necessary for nucleation and crystal growth. Additionally, the competition between water and DMSO molecules for interaction sites on the surface of biomolecules can determine solubility and aggregation tendency. This study on the stabilization of glycine in DMSO using computational methods seeks to contribute to a deeper understanding of these molecular interactions, with potential applications in the rational design of crystallization conditions. The results could provide molecular bases to explain experimental observations about the behavior of amino acids and peptides in mixed water-DMSO media, frequently used in crystallization protocols. Although there are multiple studies on different amino acids, including glycine, in water solvent, we have not found any studies on the stabilization of glycine with DMSO action.

In this study, we aim to determine the minimum number of molecules needed to stabilize the zwitterionic form of glycine in a DMSO environment using quantum mechanical methods. To validate the methodology used, we will also revisit the stabilization with water. The research compares implicit and explicit solvation models to evaluate the differences in conformational stabilization. By sequentially adding DMSO and water molecules to the glycine system in the gas phase, we seek to understand the specific solvation mechanisms and their effects on the conformational preference of the amino acid. We also analyze the specific interactions (hydrogen bonds, dipole–dipole interactions) that contribute to conformational stabilization through QTAIM (Quantum Theory of Atoms in Molecules) and NCI (non-covalent interactions) analyses.

## 2. Results

### 2.1. Conformational Search

For each molecular system, a comprehensive survey of potential energy minima was conducted using Merck Molecular Force Fields (MMFFs) [31] within the framework of rapid molecular mechanics (MM) methods, specifically “Large scales Low Mode” and Monte Carlo-based search algorithms [32].

**Scheme 1:**
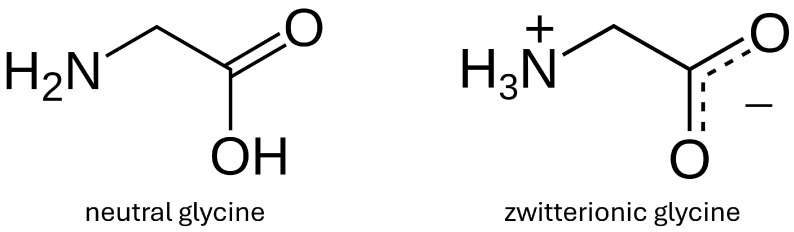
Skeletal formula of canonical/neutral glycine (**left**) and zwitterionic glycine (**right**).

Due to glycine (Gly) having several possible rotations (see Scheme 1) that can lead to different conformations, and the relative position of water molecules when interacting between different molecules influences the number of conformers, a high number of conformations will be obtained in some searches. Because the observed energetic order in MM after quantum chemical calculations will be important (vide infra), we used a nomenclature based on several parts:

N-C/ZTp-nMX

Where:

- N is the energetic position of the conformer relative to the global minimum starting from 1.
- In the case of a canonical structure, C will be used, and Z will be used when it is zwitterionic instead.
- Tp is the type of intramolecular interaction of glycine. There are three possible types of interactions, ranging from I to III and planar (p) or non-planar (np). A detailed description is given in Section 2.2.
- n is the number of molecules besides glycine that form the cluster.
- M is the molecule that will form the cluster, either water (W) or DMSO.
- X is the conformer number ordered by stability as determined by molecular mechanics.

For example, 3-CIIIp-2DMSO-13 will refer to the third (3) most stable structure by Quantum Mechanics methods (so it is easy to find in any figure) for the canonical form of glycine (C) that is based on the structure of type III (planar) glycine. It is a cluster with two DMSO molecules and, according to molecular mechanics methods, is the 13th energetical structure with respect to the global minimum (so it is easy to evaluate the performance of MM and find its Cartesian coordinates).

In Table 1, we summarize the number of structures obtained in this work using molecular mechanics for each system studied.

We encourage the reader to visit the Supplementary Materials for the .xyz files of the optimized structures of all systems studied in this work.

### 2.2. Isolated Canonical Glycine

Initially, we evaluated the conformational landscape of glycine in its canonical form. Since it is a simple system characterized by several spectroscopic techniques, including high-precision structural techniques such as rotational spectroscopy, we began by evaluating the methodology to adequately describe this molecule and its clusters. Additionally, it serves as the starting point for categorizing all the clusters obtained in this work into groups based on the intramolecular interaction in glycine.

**Figure 1:**
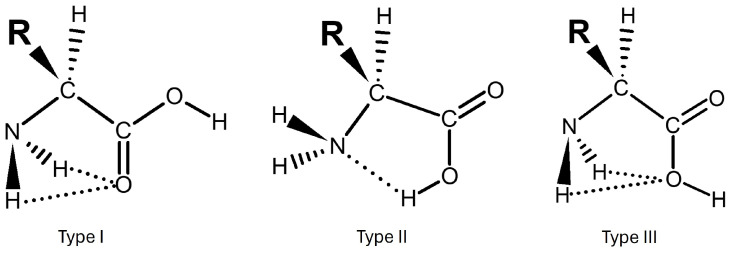
The possible configurations of α-amino acid skeleton associated with different intramolecular hydrogen bonds.

Ching-Han Hu et al. [33] provided an excellent introduction to the importance of Gly, since it generates different conformers by forming the *cis* carboxylic functional group. We will follow the labelling system for amino acids in the current literature. This way, glycine displays three different types of intramolecular hydrogen bonding: type I is a *cis* carboxyl-amino functional group, as can be seen in Figure 1 (N-H•••O=C). Type II shows a *cis* disposition between the hydroxyl and the amino groups involving hydrogen bonding between the nitrogen lone pair and the carboxylic hydrogen and resulting in a five-membered ring (O-H•••N). Type III incorporates bifurcated arrangements between the hydrogen atoms on the amino group and the oxygen atom on the hydroxyl group (N-H•••O-H). Therefore, all the clusters in the next sections will be categorized according to the intramolecular type of interaction of bare glycine: Gly Ip, Gly IIp, and Gly IIIp when bifurcated (planar) and, when not bifurcated, Gly Inp, Gly IInp, and Gly IIInp. There are more unstable types, but they are not relevant as they appear high in energy and have not been observed experimentally. For all types and more information, we recommend the excellent paper by Hong-Wei Ke et al. [34], which summarizes all work conducted until 2008. Another important study that highlights the complexity of even a simple molecule, such as glycine, was conducted by Vincenzo Barone et al. [35].

**Figure 2:**
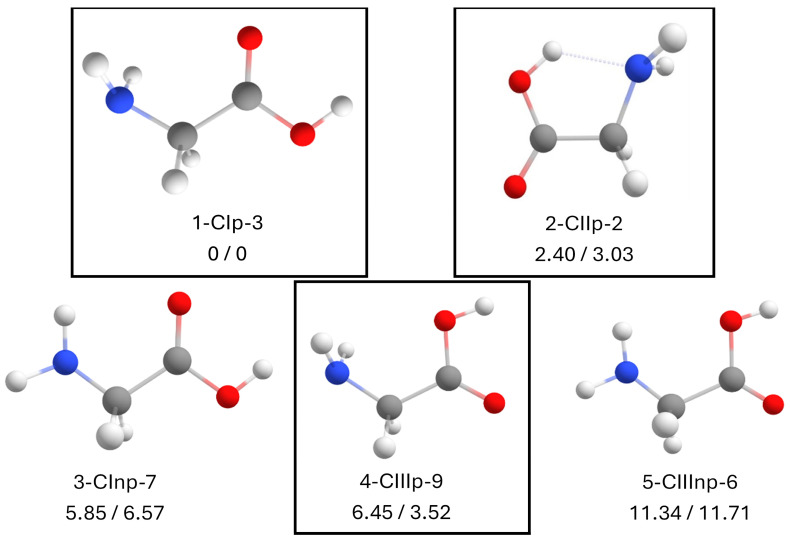
Most stable structures of canonical glycine in isolated conditions. Relative electronic energies considering the zero-point energy and relative Gibbs free energies at room temperature calculated using B3LYP-D3BJ/6-311++G(d,p) are also indicated (ΔE_ZPE_/ΔG). Values are given in kJ/mol. Black frames indicate the experimentally detected structures. Color code: Nitrogen, oxygen, carbon, and hydrogen atoms are represented by blue, red, gray, and white spheres, respectively.

From the conformational analysis, we obtained nine initial structures with molecular mechanics using a 30 kJ/mol energy filter. After optimizing the structures using B3LYP-D3BJ/6-311++G(d,p) and eliminating redundant structures, we obtained five distinct conformers (see Figure 2). The most stable conformer is 1-CIp-3. In this structure, the high electron density in the vicinity of the carbonyl oxygen facilitates the formation of hydrogen bonds with the H atoms of the amino group. Therefore, the most important stabilizing non-covalent interaction is the double N-H•••O=C interaction, and hence it is type I.

The second most stable conformer, 2-CIIp-2, has the hydroxyl group in *cis* position with respect to the amino group, but in this case, it is the hydrogen of the hydroxyl that forms the hydrogen bond with the nitrogen of the amino group through an O-H•••N hydrogen bond and is known as type II. This conformer is very close in energy to the most stable conformer (ΔE_ZPE_ = 2.40 kJ mol^−1^). Interestingly, we anticipate that this structure will lose its relevance beyond the isolated molecule for any other cluster studied in this work.

The fourth conformer, 4-CIIIp-9, is the same as the initial conformer (1-CIp-3), but this time the amino group interacts with the hydroxyl group instead of the carbonyl. In this case, the electron density is more delocalized in the hydroxyl functional group than in the carbonyl, which destabilizes the molecule about 6.45 kJ mol^−1^. This base structure will gain more relevance throughout the work and will give rise to the group/type III, according to the literature.

The last type of stable conformations we observe is based on the previous types but does not have a symmetry plane. For the third conformer, one of the hydrogens of the amine forms a hydrogen bond with the oxygen of the carboxyl (3-CInp-7), and it is considerably less stable (ΔE_ZPE_ = 5.85 kJ mol^−1^) than the global minimum and has an energy similar to that of 4-CIIIp-9. The fifth conformer also does not have a symmetry plane, and one of the hydrogens of the amine forms the hydrogen bonds with the hydroxyl group (5-CIIInp-6). In this case, we can see how the energy difference between this conformer and the most stable one is much higher (ΔE_ZPE_ = 11.34 kJ mol^−1^) due to placing the hydroxyl in *cis* position with respect to the amine. In other words, the molecule is more stabilized having the two hydrogen atoms of the amine interacting with the carbonyl group than obtaining a single bond, although more directional. These types of structures will fall within type I, II, and III, but based on the non-planar glycine and will always be less stable than the bifurcated interaction of the amino group. For these non-planar conformers, we will use the Inp, IInp, and IIInp labelling.

The results for the most stable conformers agree very well with those published in the literature, as structures 1-CIp-3, 2-CIIp-2, and 4-CIIIp-9 have been detected experimentally [36,37,38,39] in good agreement with the relative free energies in Figure 2 and Table S1. Moreover, the rotational constants of Gly-I and Gly-II have been characterized by microwave spectroscopy [36,37,38] so the performance of B3LYP-D3BJ/6-311++G(d,p) regarding the fidelity of the calculated structures can be assessed. For 1-CIp-3, the calculated values using B3LYP-D3BJ/6-311++G(d,p) differ by less than 0.02, 0.71, and 0.53% for the rotational constants of *A*, *B*, and *C*, respectively. Additionally, for 2-CIIp-2, they differ by 0.26, 0.23, and 0.02% for the rotational constants of *A*, *B*, and *C*, respectively. Finally, we did not find any discussion about the non-detection of the 3-CInp-7 structure in the literature, which, if 4-CIIIp-9 has been detected, could also be detected. The obvious explanation is that 4-CIIIp-9 is less populated than 3-Cinp-7 if considering the free Gibbs energies. Additionally, we performed a relaxed potential energy surface (PES) scan of 3-Cinp-7, rotating the C-C-N-H dihedral angle (see Figure S2) and found that there is a low interconversion barrier (0.72 kJ mol^−1^) into the type I configuration, which usually occurs in supersonic expansions with a barrier height below 4.8 kJ mol^−1^ [40,41].

Thus, we can conclude that the calculation level used, B3LYP-D3BJ/6-311++G(d,p), constitutes a good compromise between quality of results and computational cost. All the structures are shown in Figure S1, while Table S1 shows relevant spectroscopic parameters.

The reason for not obtaining the other type of interactions beyond Types I to III, as found in the literature [33,34], is due to the energy cut-off during the conformational search. In any case, those forms are very high in energy and not relevant to the current work.

### 2.3. Canonical Glycine with 1 Water Molecule

**Figure 3:**
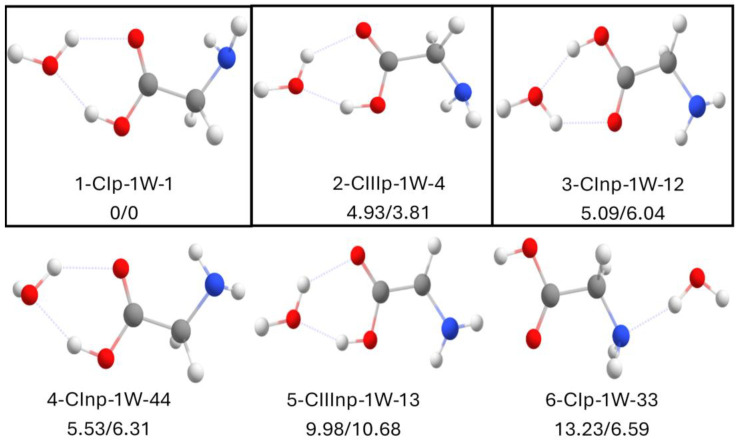
Most stable structures of canonical glycine and one water molecule in isolated conditions. Relative electronic energies considering the zero-point energy and relative Gibbs free energies at room temperature calculated using B3LYP-D3BJ/6-311++G(d,p) are also indicated (ΔE_ZPE_/ΔG). Values are given in kJ/mol. Black frames indicate the experimentally detected structures. Color code: Nitrogen, oxygen, carbon, and hydrogen atoms are represented by blue, red, gray, and white spheres, respectively.

The conformational search led to 47 initial conformers. After optimizing the structures and eliminating redundant ones, we obtained 24 distinct conformers. The first important observation we can make is the enormous aggregation power of water and how the water molecule is a great stabilizer, since, despite the large number of structures, there are only five very stable structures below 13.3 kJ mol^−1^. The optimized structures at the B3LYP-D3BJ/6-311++G(d,p) level of theory are collected in Figure 3. All of them are based on types I and III of the bare glycine. In all the stable structures, the water molecule forms two hydrogen bonds with the carboxylic groups of glycine, where water acts as a proton donor to the carboxyl group of glycine (O-H•••O=C) and as a proton acceptor to the hydroxyl group of glycine (O-H•••O), closing the intermolecular ring. Among them, type I glycine is the global minimum (1-Cip-1W-1), while type III (2-CIIIp-1W-4) is less stable (4.93 kJ mol^−1^), but is now the second most stable structure, and its stability with respect to type I structure has increased ≈1.5 kJ mol^−1^ with respect to bare glycine. Nevertheless, the energy difference between the most stable structure and 2-CIIIp-1W-4 explains why only the global minimum has been observed using microwave spectroscopy [42]. On the other hand, other techniques, such as the jet-cooled spontaneous Raman spectrum, have detected the 2-CIIIp-1W-4 and 3-Cinp-1W-12 structures [43], but their signals were weak, which agrees with the calculated relative energies. Regarding the 3-Cinp-1W-12 structure, we conducted another relaxed PES scan rotating the C-C-N-H dihedral angle, and, as Figure S4 shows, it again relaxes into the global minimum, so we are surprised about its detection using jet-cooled experiments. This highlights how even a simple molecule such as glycine is a complicated system, and it could be a good idea to revisit the microwave spectrum of glycine–water complex using recent advances [44,45] in order to look for more candidates. Finally, for the conformer characterized by microwave spectroscopy [42], 1-Cip-1W-1, the rotational constants computed at the B3LYP-D3BJ/6-311++G(d,p) level differ by less than 0.13, 1.08, and 1.00% for the rotational constants of *A*, *B*, and *C* using B3LYP-D3BJ/6-311++G(d,p) from the experimental *A*, *B*, and *C* rotational constants, confirming that this methodology provides reliable results.

Therefore, in the case of the Gly-H_2_O cluster, we see that the most stabilized structures resemble those of type I and III free glycine. The biggest difference is the absence of the base structure of type II glycine in the most stable conformers due to the high stability provided by the two hydrogen bonds contributed by water, in addition to the N-H•••O=C interaction, which leads to the destabilization of the arrangement in which the hydroxyl group of glycine forms hydrogen bonds with the amino group. In fact, type II structures appear at a relative energy of ≈ 13 kJ mol^−1^ with respect to the global minimum (see Figure S3 in the Supplementary Materials).

Again, we can see how the B3LYP-D3BJ/6-311++G(d,p) methodology provides good results and explains the experimental findings. All the optimized structures are shown in Figure S3, while Table S3 shows relevant spectroscopic parameters.

### 2.4. Canonical Glycine with 2 Water Molecules

**Figure 4:**
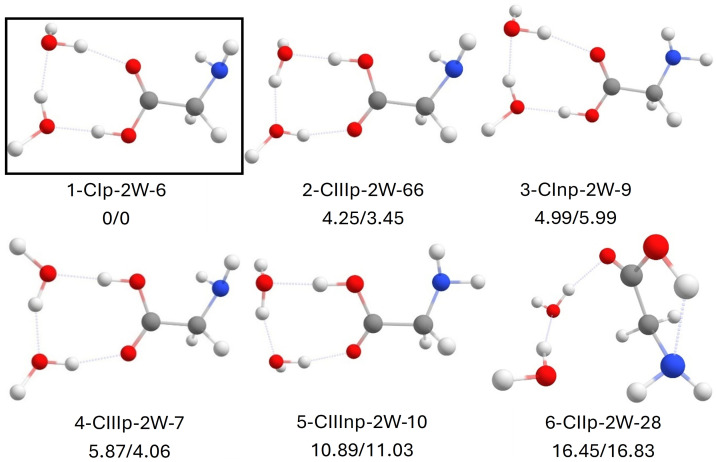
Most stable structures of canonical glycine and two water molecules in isolated conditions. Relative electronic energies considering the zero-point energy and relative Gibbs free energies at room temperature calculated using B3LYP-D3BJ/6-311++G(d,p) are also indicated (ΔE_ZPE_/ΔG). Values are given in kJ/mol. Black frames indicate the experimentally detected structures. Color code: Nitrogen, oxygen, carbon, and hydrogen atoms are represented by blue, red, gray, and white spheres, respectively.

In this case, the conformational search provided us with a larger number of conformers of the system formed by glycine and two water molecules. Specifically, a total of 71 conformers were obtained, which, after optimizing and eliminating redundant structures, were reduced to 37 distinct conformers. Figure 4 collects the most stable structures below 16.5 kJ mol^−1^.

The observations made in the complex with one water molecule can be extrapolated to this system with two water molecules. The Gly-2H_2_O cluster forms rings of intramolecular interactions formed by three highly stabilizing hydrogen bonds supported by the carboxyl group. We see that even in the less stable conformations, water always forms hydrogen bonds with itself, which is predictable due to the strong cohesion of this solvent. All the structures below 15 kJ mol^−1^ are, again, based either on type I or type III configuration of bare glycine, with type I being the global minimum (structure 1-Cip-2W-6). The most stable structure of type III, 2-CIIIp-2W-66, this time differs in having less relative energy (ΔE_ZPE_ = 4.25 kJ mol^−1^) with respect to the global minimum type I, which is almost a 15% reduction in relative energy with respect to type I. It seems that, as the number of water molecules increases, there will be more isoenergetic arrangements. In any case, there is still a considerable difference between type I and III-based structures, and the results confirm again why only the global minimum has been detected in the gas phase [46]. Finally, the rotational constants of the detected 1-Cip-2W-6 by microwave spectroscopy differ by less than 2.49, 0.64, and 1.05% for the rotational constants of *A*, *B*, and *C* using B3LYP-D3BJ/6-311++G(d,p), confirming that, despite the floppiness of this molecule, this methodology provides good results.

Type II structures, such as 6-CIIp-2W-28, appear at a relative energy of >16 kJ mol^−1^ with respect to the global minimum.

All the optimized structures are shown in Figure S5, while Table S3 shows relevant spectroscopic parameters.

### 2.5. Zwitterionic Glycine with 1 Water Molecule

For the aggregate of the zwitterionic glycine molecule with one water molecule, the B3LYP-D3BJ/6-311+G(d,p) calculation level did not lead to a stable structure. After optimization, glycine always spontaneously returned to its canonical state. That is, the hydrogens of the ammonium group (-NH_3_^+^) initially (zwitterion) form a hydrogen bond with the alkoxide group (-O^−^), but, upon optimization, the proton is transferred from the amino group to the hydroxyl group.

The initial bibliography argued that one water molecule is enough to stabilize the zwitterionic structure of glycine [23]. Reviewing the literature, we found that some authors argued that it is a matter of computational methodology [25]. As it is critical for this work to find an appropriate methodology, we could not ignore this fact and tried to perform computational calculations at different calculation levels using different bases to solve the puzzle.

For all the conformers explored, the zwitterionic form of glycine with one water molecule spontaneously relaxes to a complex of glycine in its canonical form and water with various methodologies and basis sets, including B3LYP, B3LYP-D3BJ, B2PLYP-D3BJ, MP2, M062X, and ωB97X-D with the 6-311++G(d,p) basis set.

On the other hand, we were able to stabilize the zwitterionic system with one water molecule if we used the Hartree–Fock method regardless of the basis set or if we employed the B3LYP-D3BJ functional with a poorer basis (6-31+G(d)). These results suggest that the optimization of the zwitterionic form of glycine with one water molecule is “fictitious” when using inadequate methodology that does not include electron correlation, such as the HF methodology, or a low-quality basis that describes molecular orbitals imprecisely.

### 2.6. Zwitterionic Glycine with 2 Water Molecules

**Figure 5:**
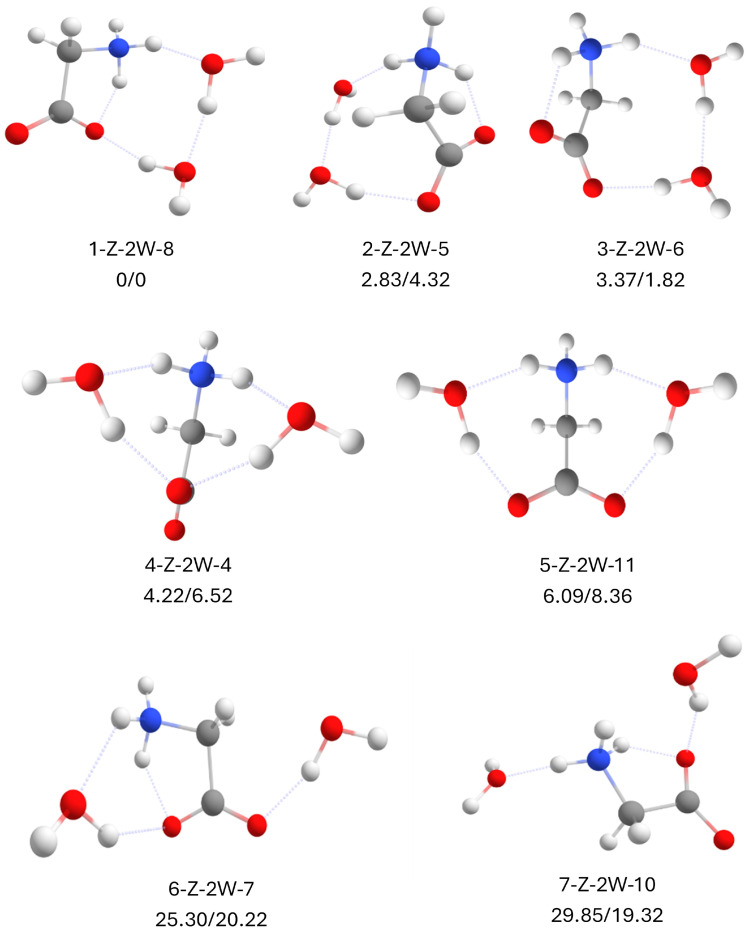
Most stable structures of zwitterionic glycine and two water molecules in isolated conditions. Relative electronic energies considering the zero-point energy and relative Gibbs free energies at room temperature calculated using B3LYP-D3BJ/6-311++G(d,p) are also indicated (ΔE_ZPE_/ΔG). Values are given in kJ/mol. Color code: Nitrogen, oxygen, carbon, and hydrogen atoms are represented by blue, red, gray, and white spheres, respectively.

Finally, and to finish with the glycine–water system at an explicit level, we pursued the system of glycine in its zwitterionic form with two water molecules. Unlike the system with a single water molecule, when incorporating two water molecules, it is already possible to obtain stable minima with the chosen methodology. Molecular mechanics provided 13 initial structures; after optimizing and eliminating redundant ones, 7 distinct structures remained, as shown in Figure 5.

The results indicate several key findings. On the one hand, there is a decrease in the number of conformers observed, even compared to canonical glycine with one water molecule. This is due to the charge of the ammonium group (NH_3_^+^). The existence of this positive charge will allow those structures in which the ammonium group (NH_3_^+^) interacts with the lone pairs of the oxygen of the carbonyl group or water to be stabilized. On the other hand, this leads to asymmetric structures being the most stable, due to the high cohesion of water stabilizing more when these molecules interact with each other than when they do only with glycine.

Here, characterizing the structures as type I or III makes no sense, as the hydrogen from the hydroxyl group is lost. However, we can divide the structures into four large groups: one group accounts for those structures where water self-aggregates starting from the NH_3_^+^ group and ending at the oxoanion groups (C=O^−^), accepting two intermolecular hydrogen bonds, that is, forming a cooperative network of hydrogen bonds N-H•••O-H•••O-H•••O=C, with the same oxoanion group being a proton acceptor due to the intramolecular N-H•••O=C hydrogen bond. The global minimum (1-Z-2W-8) is based on this configuration. In the second group, there is also a cooperative network of hydrogen bonds N-H•••O-H•••O-H•••O=C, but in this case, it is the other oxoanion group that acts as a proton acceptor of the intramolecular N-H•••O=C hydrogen bond, such as the second most stable structure 2-Z-2W-5 or 3-Z-2W-6. The latter group is 2.83 kJ mol^−1^ less stable.

The third large group is where water molecules do not interact with each other, but each water molecule acts as a proton acceptor to the NH_3_^+^ group and to either the same oxoanion group (4-Z-2W-4) or a different oxoanion group (5-Z-2W-11). These structures lose more than 4 kJ/mol of stability, confirming the power of water to self-aggregate.

Finally, the fourth group collects structures where one water molecule interacts with the NH_3_^+^ or the C=O group, but the other water molecule has weak intramolecular interactions such as C-H•••O-H interactions. These structures are highly unstable and appear at a relative energy of >25 kJ mol^−1^.

All the optimized structures are shown in Figure S6, while Table S4 shows relevant spectroscopic parameters.

### 2.7. Canonical Glycine with 1 DMSO Molecule

For DMSO, as is obvious, no conformational search was performed, as there is only one structure, so we proceeded directly with the DMSO complexes. The results are exciting since, a priori, it is difficult to intuit what will happen when introducing a polar aprotic solvent to a glycine molecule with various functional groups.

**Figure 6:**
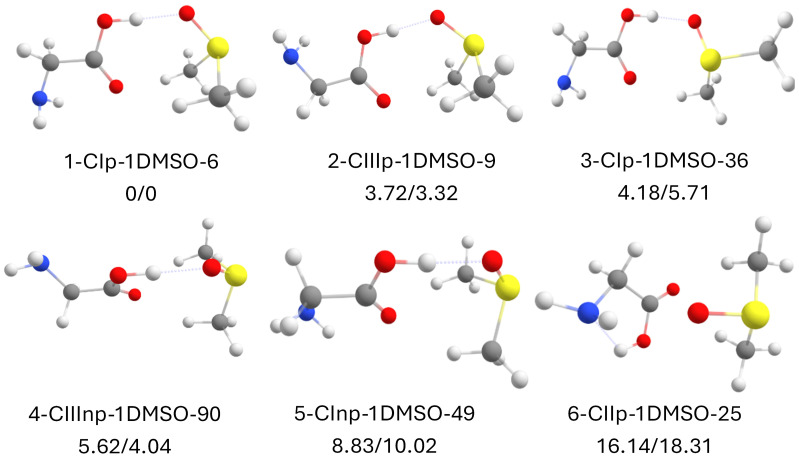
Most stable structures of canonical glycine and one DMSO molecule in isolated conditions. Relative electronic energies considering the zero-point energy and relative Gibbs free energies at room temperature calculated using B3LYP-D3BJ/6-311++G(d,p) are also indicated (ΔE_ZPE_/ΔG). Values are given in kJ/mol. Color code: Nitrogen, oxygen, sulfur, carbon, and hydrogen atoms are represented by blue, red, yellow, gray, and white spheres, respectively.

As mentioned earlier, for the glycine–DMSO system, we obtained a total of 110 initial conformers, which, after optimizing and eliminating redundant structures, yielded a total of 22 distinct conformers. Figure 6 collects the six most stable structures below ≈ 16 kJ mol^−1^. Similar to glycine with one water molecule, the |glycine + DMSO| system leads to a clear global minimum whose structure is again based on the most stable structure of glycine (type I, 1-cIp-1DMSO-6). How does DMSO aggregate? Calculations indicate that DMSO uses an optimal chemical strategy to maximize its stability, which will be relevant to discuss later: on the one hand, like water, the oxygen of the sulfoxide group acts as a proton acceptor (S=O•••H-O). However, being aprotic, it solves the stability problem by using a more forgotten functional group among the possible hydrogen bond formers: the methyls (more specifically, the C-H group). Despite carbon not being a very electronegative atom in general and its hydrogen bonds not being very strong, DMSO incorporates in such a way that the two methyl groups interact as proton donors to the carbonyl group of glycine. By adding two hydrogen bonds, one for each methyl, they compensate for the lack of strength of this intramolecular bond. This will be much more visual with the QTAIM and NCI calculations (vide infra).

The global minimum is followed by a structure with the same intra- and intermolecular interactions, but with the acid group rotated 180°, i.e., based on type III glycine (2-CIIIp-1DMSO-9). This structure is 3.7 kJ mol^−1^ less stable than the global minimum. We note that type III-based structures are now 2.5 kJ/mol lower in energy relative to type I structures in glycine or 1 kJ mol^−1^ lower relative to type I in |glycine + water|. The rest of the structures are based on these types but with a single methyl group interacting with the C=O group, such as in 3-cIp-1DMSO-36 at >4 kJ mol^−1^, or adopting more restrained configurations. Finally, it is worth mentioning that type II structures are more than 16 kJ mol^−1^ less stable than the global minimum, such as 6-CIIp-1DMSO-25.

All the structures are shown in Figure S7, while Table S5 shows relevant spectroscopic parameters.

### 2.8. Canonical Glycine with 2 DMSO Molecules

**Figure 7:**
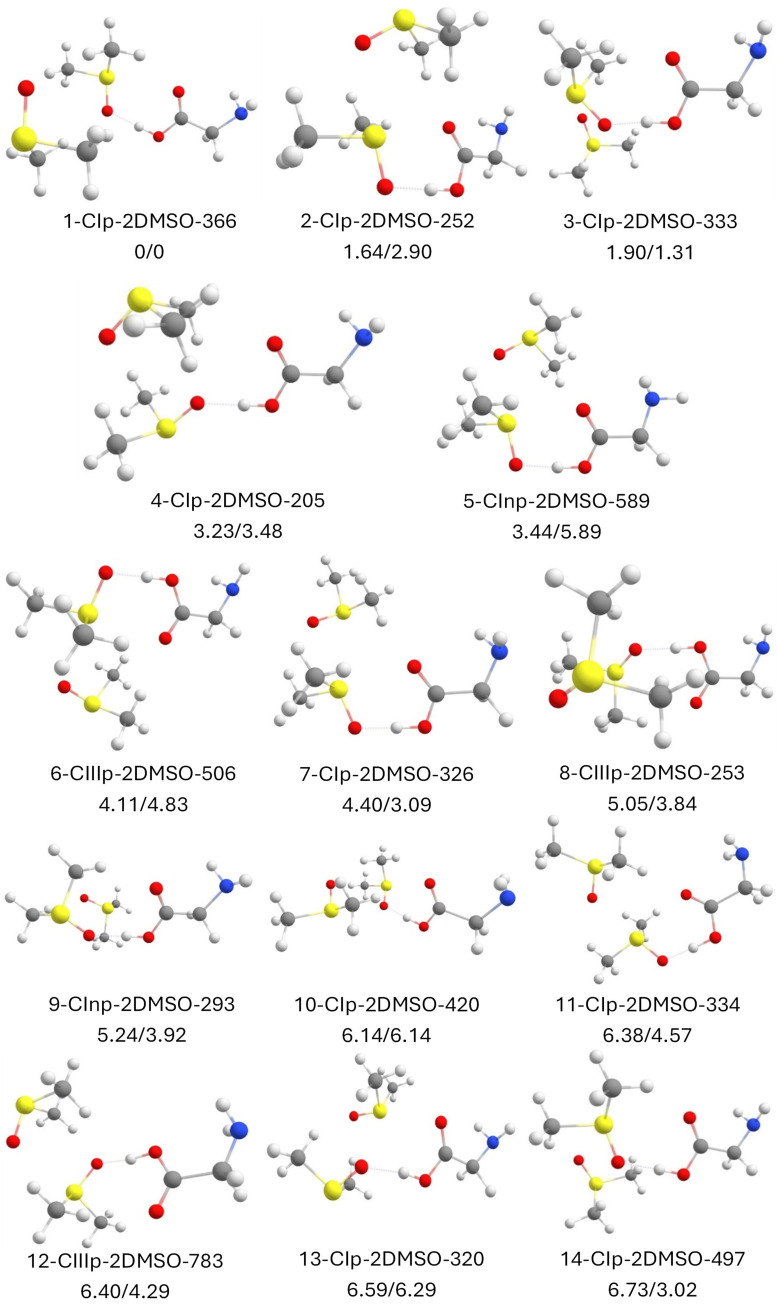
Most stable structures of canonical glycine and two DMSO molecules in isolated conditions. Relative electronic energies considering the zero-point energy and relative Gibbs free energies at room temperature calculated using B3LYP-D3BJ/6-311++G(d,p) are also indicated (ΔE_ZPE_/ΔG). Values are given in kJ/mol. Color code: Nitrogen, oxygen, sulfur, carbon, and hydrogen atoms are represented by blue, red, yellow, gray, and white spheres, respectively.

In this case, the conformational search gave us 616 conformers. After eliminating redundant conformers, we were left with a large number of 246 distinct conformers. Figure 7 collects the most stable structures below ≈ 7 kJ mol^−1^. We observe a considerable increase in the number of conformers compared to the glycine and two water molecules system. This is due to what has already been mentioned about the great self-aggregation power of water, which results in more selective structures, while DMSO generates more disorganized structures (at least for structures higher in energy).

The results obtained are very illustrative because, again, we can classify the obtained structures into two large groups: those that are based on type I of glycine, such as the five most stable structures—1-cIp-2DMSO-366, 2-cIp-2DMSO-252, 3-cIp-2DMSO-333, 4-cIp-2DMSO-205, and 5-cInp-2DMSO-589—and those based on type III. Curiously, again, the most stable structure based on type III (6-CIIIp-2DMSO-506) is found at about 4.11 kJ mol^−1^ with respect to the global minimum.

The most stable structures can be considered those where a DMSO molecule aggregates to the complex formed by glycine and the other DMSO molecule. Specifically, the most stable cluster is formed from the 1-cIp·1DMSO-56 structure that we have commented on, glycine+DMSO is strongly stabilized by an O-H•••O=S intermolecular hydrogen bond, where the new DMSO incorporates on the DMSO side to form a very stable dimer with itself. This last aggregation is very striking for its symmetry since the two methyls of one DMSO molecule interact in a bifurcated manner with the O=S group, respectively. Additionally, both molecules’ methyl groups interact with the O=S group of the other molecule. This gives an interesting idea to address the study of DMSO homomers in the future. In summary, the most stable structure of glycine with two DMSO molecules is one that achieves a DMSO dimer, while one of these DMSO molecules aggregates to glycine in a way that resembles one of the most stable structures of glycine–DMSO.

The second most stable structure is very similar to the most stable one. However, this time, the second DMSO unit, instead of maximizing its contact via the methyl groups with the other DMSO molecule, has the methyls interacting via intermolecular hydrogen bonds in a bifurcated manner with the carbonyl of glycine.

The following structures are small variations of the first two and therefore have a similar relative energy. Similarly, when glycine adopts type III at a relative energy of ≈4 kJ mol^−1^, we will also find similar structures based on this group. Type II structures, on the other hand, appear > 10 kJ mol^−1^ above relative to the global minimum.

All the structures are shown in Figure S8, while Table S6 shows relevant spectroscopic parameters.

### 2.9. Zwitterionic Glycine with 1 DMSO Molecule

Next, we begin with the complexation of DMSO molecules with the zwitterionic form of glycine, starting with one DMSO molecule.

**Figure 8:**
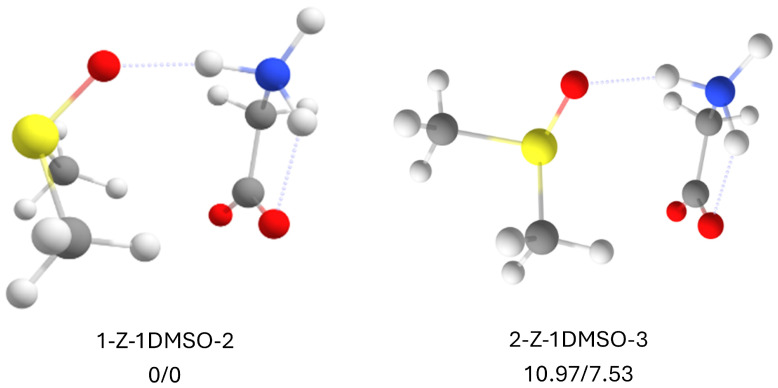
Most stable structures of zwitterionic glycine and one DMSO molecule in isolated conditions. Relative electronic energies considering the zero-point energy and relative Gibbs free energies at room temperature calculated using B3LYP-D3BJ/6-311++G(d,p) are also indicated (ΔE_ZPE_/ΔG). Values are given in kJ/mol. Color code: Nitrogen, oxygen, sulfur, carbon, and hydrogen atoms are represented by blue, red, yellow, gray, and white spheres, respectively.

As with the water system, the zwitterionic form leads to a reduction in the number of conformers. Thus, for the zwitterionic glycine and DMSO complex, we obtained only four conformers from the molecular mechanics search, which, after optimizing and eliminating redundant structures, provide only two distinct conformers. Figure 8 collects the two stable structures. The first thing observed is that, when performing the stabilization of the zwitterionic glycine molecule with a single DMSO molecule, unlike with water, DMSO does stabilize the zwitterionic structure after performing the optimization through DFT.

The way in which the molecular system is stabilized is quite impactful—although once seen, it is somewhat logical—which reflects the importance of computational chemistry. On one hand, without a carboxylic acid group (-COOH) but with two equivalent oxoanion groups (C=O^−^) with the negative charge delocalized in the carboxylate group (COO^−^), the two methyls are oriented to this electron density of both oxoanions, as can be seen for the global minimum 1-Z-1DMSO-2. In turn, the NH_3_^+^ group acts as a proton donor to the O=S group, closing the cycle of intramolecular interactions. The second structure, 2-Z-1DMSO-3, is the same but sacrifices the double interaction of the two methyls with the two oxoanions, so that only one of the methyls, although more directionally, interacts with the oxoanion group. The loss of an interaction destabilizes the complex by almost 11 kJ mol^−1^.

All the structures are shown in Figure S9, while Table S7 shows relevant spectroscopic parameters.

### 2.10. Zwitterionic Glycine with 2 DMSO Molecules

**Figure 9:**
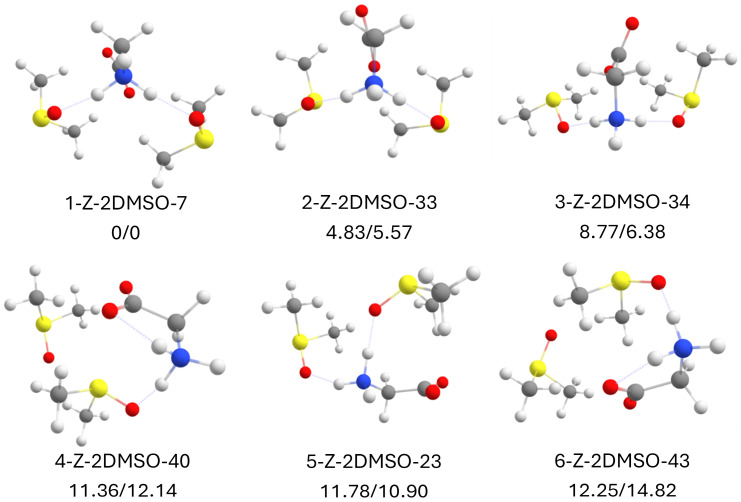
Most stable structures of zwitterionic glycine and two DMSO molecules in isolated conditions. Relative electronic energies considering the zero-point energy and relative Gibbs free energies at room temperature calculated using B3LYP-D3BJ/6-311++G(d,p) are also indicated (ΔE_ZPE_/ΔG). Values are given in kJ/mol. Color code: Nitrogen, oxygen, sulfur, carbon, and hydrogen atoms are represented by blue, red, yellow, gray, and white spheres, respectively.

The following system of molecular aggregates to study is the zwitterionic form of glycine with two DMSO molecules. Molecular mechanics provided us with 45 structures, which, after optimization and elimination of redundant structures, resulted in 17 distinct structures. Figure 9 collects the most stable structures below 12.5 kJ mol^−1^.

Regarding the obtained structures, the first important result is that the introduction of two DMSO molecules leads to a reduction in the energy gap between the most stable structures. In the most stable structure, 1-Z-2DMSO-7, now two of the hydrogens of the NH_3_^+^ group act as donors, each to the O=S group of each DMSO molecule, while the methyl groups of each DMSO interact with the oxoanion groups of glycine. It is curious how, again, the system contemplates asymmetric structures as more stabilized. In this case, it is the oxoanion group that breaks the symmetry.

The second most stable structure, 2-Z-2DMSO-33, is similar to the global minimum. However, instead of the four methyl groups interacting via intermolecular hydrogen bonds to the oxoanion group, one such interaction is lost so that one methyl group is now positioned to interact with the oxoanion group and the O=S group at the same time, resulting in an almost 5 kJ mol^−1^ decrease in stability.

The rest of the structures are quite similar, although with different relative positions, which minimizes the inter and intramolecular interactions. In any case, most of the structures of zwitterionic glycine and DMSO are stabilized through the interactions between the NH_3_^+^ group to the O=S group, and through the interactions between the methyl groups and the oxoanion groups of glycine.

All the structures are shown in Figure S10, while Table S8 shows relevant spectroscopic parameters.

### 2.11. Explicit Solvent Model

As we show in the previous sections, the explicit solvation indicates that DMSO is capable of stabilizing the zwitterionic form of glycine. Moreover, it does so with a single DMSO molecule, which does not occur with water. Another important piece of data would be the relative energy between the zwitterionic form with the complexes and their canonical form. Table 2 shows the results of the comparison between the most stable structures of glycine canonical versus glycine zwitterionic (C-X versus glycine Z-X: X indicates the water or DMSO complex).

As we can see in Table 2, adding up to two solvent molecules (H_2_O or DMSO) still does not manage to stabilize the zwitterionic form complex over the canonical form. On the other hand, adding one DMSO molecule seems to stabilize the complex almost equally as two water molecules. Finally, and most importantly, adding a second DMSO molecule stabilizes the zwitterionic form of the complex by a factor of ≈3 with respect to adding a single DMSO molecule, which is a very important fact, especially considering that now the zwitterionic form is at 12 kJ mol^−1^ above the most stable form of the canonical form of glycine. At these values, the zwitterionic form begins to be relevant.

Our comparative energy analysis between canonical and zwitterionic forms shows that while two DMSO molecules significantly reduce the energy gap between these forms (to approximately 12 kJ/mol), the canonical form remains more stable in the gas phase. However, the trend suggests that additional DMSO molecules might eventually invert this stability, so it would be interesting to add more DMSO molecules to the systems in future studies to test how many molecules are necessary to stabilize the zwitterionic form over the canonical form of glycine.

### 2.12. Implicit Solvent Model

So far, an explicit solvation model has been carried out. This model allows us to understand the behavior of DMSO with glycine at the molecular level. In this section, we will contrast the results obtained with implicit solvation (using a polarizable continuum model).

Table 3 shows the relative energies of the most stable canonical and zwitterionic forms of glycine in water and DMSO when using a polarizable continuum model (PCM) and the solvation model based on electron density (SMD). As can be seen, the results for water are as expected. It is well known that glycine is found in its zwitterionic form in an aqueous solution. As shown, the zwitterionic form is, according to both models, more stable in a continuous water medium than its canonical form, particularly for the SCM model. This allows us to validate our results once again. What do we find with DMSO? The results are interesting because they indicate that, like with water, the zwitterionic form in a continuous DMSO medium is more stable than its canonical form. However, if we consider the Gibbs free energy, which is more realistic, the stability is inverted for the PCM model, and the canonical form becomes the most stable, although by a small amount of energy. SMD predicts the canonical form to be more stable, but still predicts that the population of the zwitterionic form starts to be an appreciable quantity (≈13.5%).

**Figure 10:**
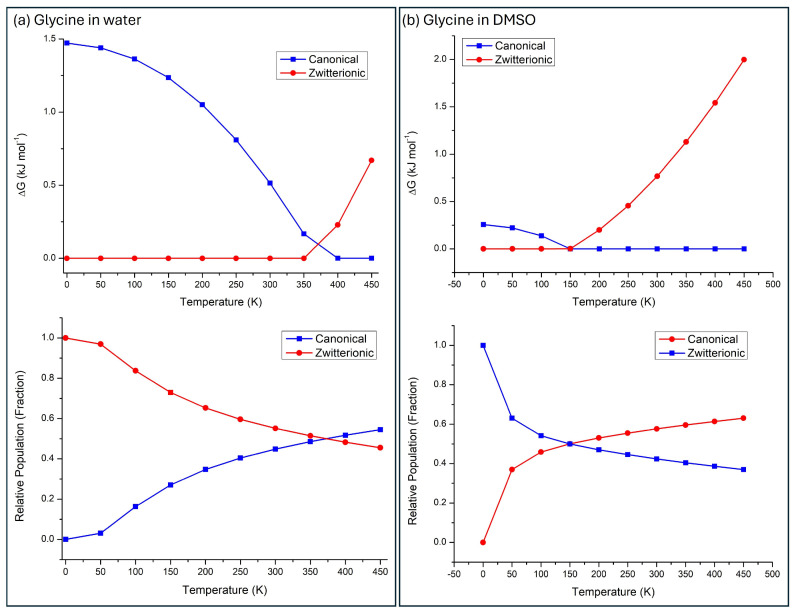
Relative Gibbs free energies (Top, kJ/mol) and population fraction (bottom) between canonical glycine and its zwitterionic form in (**a**) water and (**b**) DMSO at different temperatures (K). The calculations were performed using B3LYP-D3BJ/6-311++G(d,p). The population fraction was estimated using a Boltzmann ensemble of the two species at thermal equilibrium.

These results made us think about what happens as the temperature varies. Obviously, at T = 0 K, we should have only the enthalpy coinciding numerically with the value of the relative electronic energy with the zero-point correction. Thus, we performed the calculation of the variation of the Gibbs free energy (relative to the most stable species at each temperature). The results are shown in Figure 10. When using the PCM model, the zwitterionic glycine structure with water is generally more stable than its canonical form unless it exceeds 375 K. On the other hand, for DMSO, the canonical form is more stable if the system is above about 150 K, below which temperature the zwitterionic form begins to be more stable. We did not plot the results using the SMD model as they show a flat response of the values collected in Table 3 at T = 310 K. This information could be important to contrast the validity of both methods if experiments are performed. Moreover, we believe these results are useful for researchers who crystallize proteins since DMSO is highly employed in these cases. However, it should be noted that these results are with a 100% DMSO medium, contrary to the usual use of DMSO, which is, generally (depending on the proteins), not highly concentrated. In any case, we believe this is a starting point for understanding many of the properties of DMSO.

### 2.13. Analysis of Intramolecular Interactions: NCI and QTAIM

**Figure 11:**
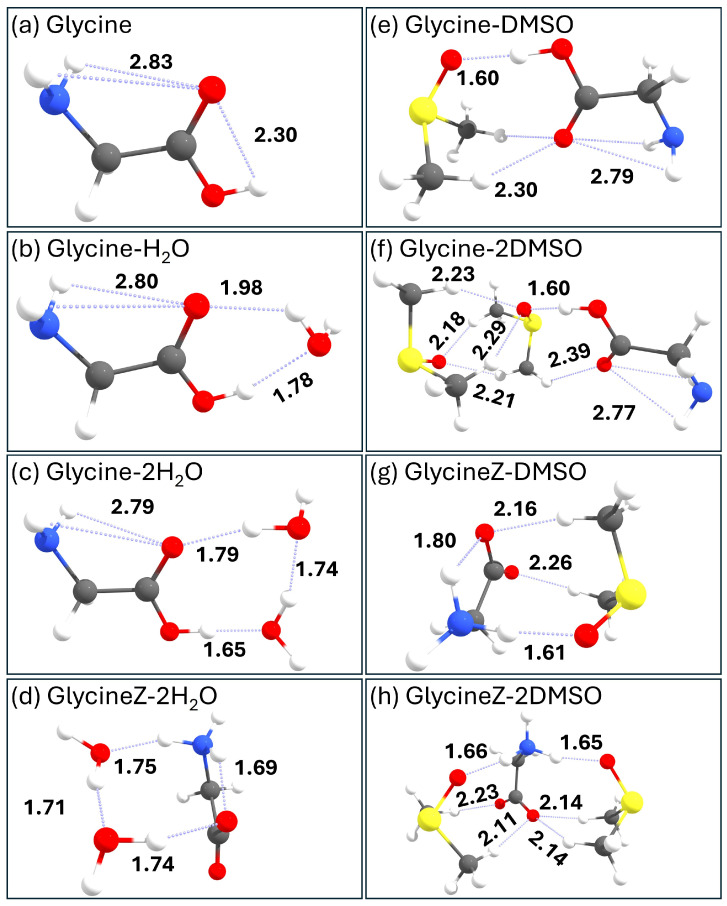
Most stable structure of each system studied in this work calculated using B3LYP-D3BJ/6-311++G(d,p): (**a**) canonical glycine; (**b**) canonical glycine and one water molecule; (**c**) canonical glycine and two water molecules; (**d**) zwitterionic glycine and two water molecules; (**e**) canonical glycine and one DMSO molecule; (**f**) canonical glycine and two DMSO molecules; (**g**) zwitterionic glycine and one DMSO molecule; (**h**) zwitterionic glycine and two DMSO molecules in isolated conditions. Bond distances in Å are indicated. Note that bare zwitterionic glycine and its cluster with one water molecule are not in the figure, as they were not optimized. Color code: Nitrogen, oxygen, sulfur, carbon, and hydrogen atoms are represented by blue, red, yellow, gray, and white spheres, respectively.

To finalize this study, we performed an analysis of the bond interactions that take place in the most stable conformers of each of the complexes presented in this work. For this purpose, we used the AIMAll program [47] to calculate the different bond parameters and obtain the molecular graph. Additionally, for obtaining non-covalent interactions, we used the NCIPlot [48,49] programs to obtain the distribution of electron density around the molecule and VMD [50] to visualize their representation. To complement the results, the most relevant bond distances are given in Figure 11 for the most stable structures of each molecular system studied in this work.

**Figure 12:**
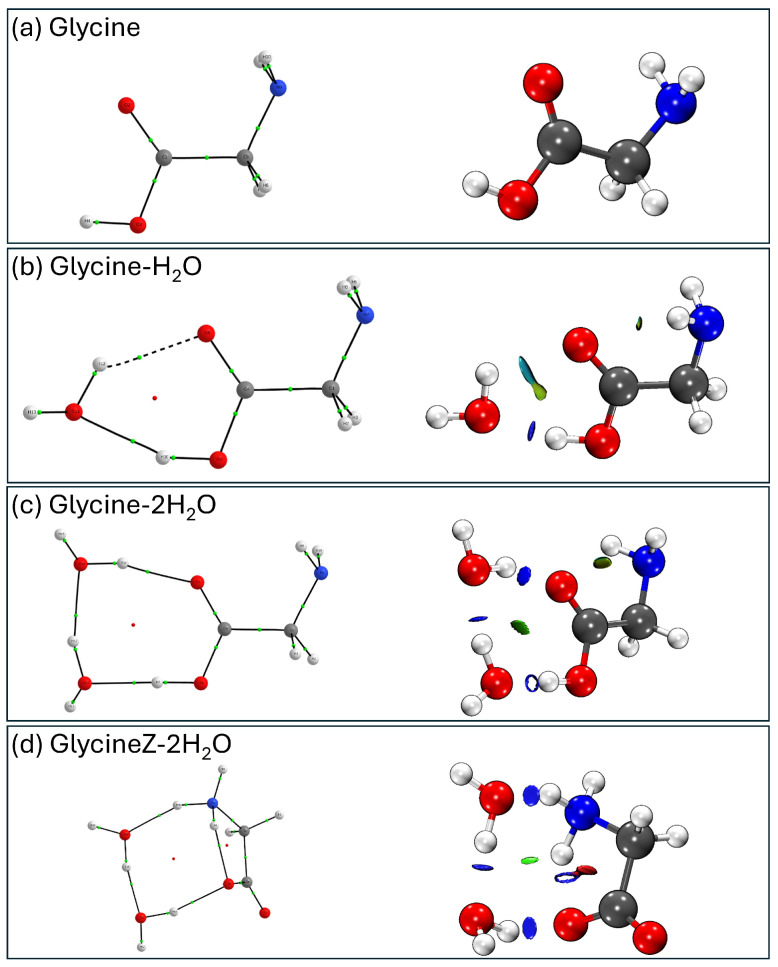
Molecular graph (**left**) and NCIPlot (**right**) of the most stable structure of (**a**) canonical glycine, (**b**) canonical glycine and one water molecule, (**c**) canonical glycine and two water molecules, (**d**) zwitterionic glycine and two water molecules calculated using B3LYP-D3BJ/6-311++G(d,p). Note that bare zwitterionic glycine and its cluster with one water molecule are not in the figure, as they did not converge. Gray corresponds to carbons, blue to nitrogen, red to oxygen, and white to hydrogen. In the AIMAll molecular graphs, the small green, red, and blue spheres represent BCPs, RCPs, and CCPs, respectively. For the NCIPlot: red surfaces correspond to repulsive forces, blue surfaces to moderate attractive forces, and green surfaces to weak attractive interactions. A contour value of 0.35 was used for the representation. Color code: Nitrogen, oxygen, carbon, and hydrogen atoms are represented by blue, red, gray, and white spheres, respec-tively.

The QTAIM analysis in Table S9 and Figure 12 reveals that all canonical glycine bonds present values characteristic of shared interactions typical of covalent bonds: high values of electron densities, negative Laplacians and total energy densities, and |V(r)|/G(r) ratios greater than 2.

For the glycine–water aggregates, the QTAIM analysis in Table S10 and Figure 12 shows that the Gly-H_2_O cluster presents a bond critical point between the carbonyl oxygen of glycine and one of the H atoms of H_2_O, with the typical characteristics of hydrogen bonds: low values of electron density, positive Laplacian and total energy density, and |V(r)|/G(r) ratio less than 1. Additionally, in glycine and one water molecule, the bond between the O atom of the water molecule and the H atom of the hydroxyl group of glycine presents a certain degree of covalency since the value of the total energy density is slightly negative and the |V(r)|/G(r) ratio is slightly higher than 1. These findings are consistent with the intermolecular distances shown in Figure 11. This is again true in canonical glycine and two water molecules, with the addition that the intermolecular interaction between water’s hydroxyl group and the O=C group of glycine (O-H•••O=C) also presents a certain degree of covalency consistent with the shorter distance of this intermolecular hydrogen bond than in Gly-H_2_O. Regarding the zwitterionic glycine and two water molecules, the same considerations can be made, but it is now the NH_3_^+^ group acting as a proton donor.

**Figure 13:**
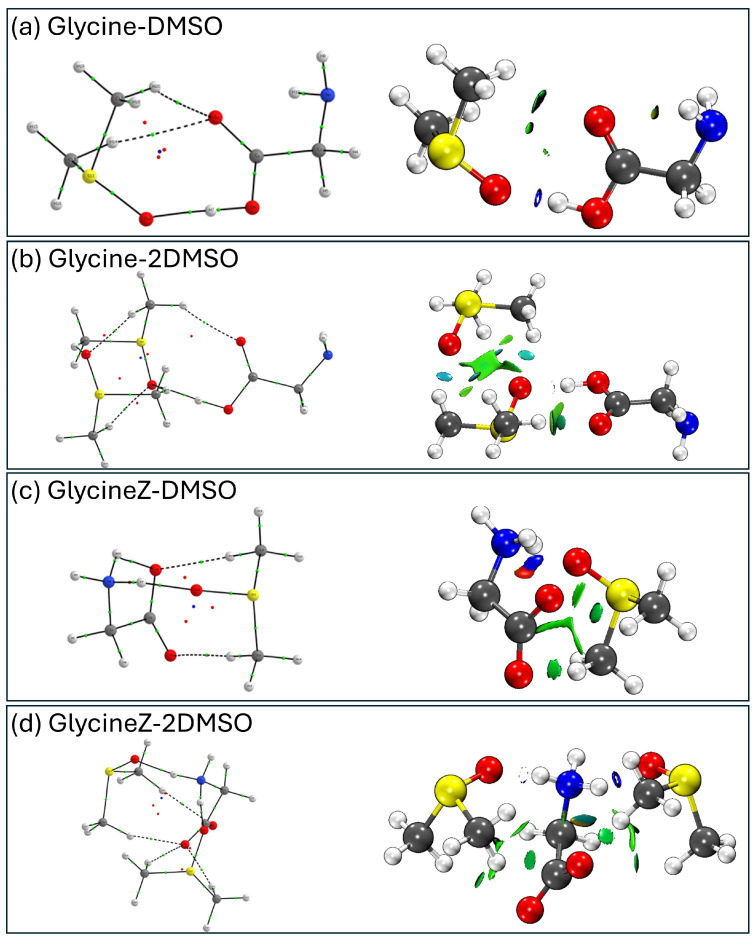
Molecular graph (**left**) and NCIPlot (**right**) of the most stable structure of (**a**) canonical glycine and one DMSO molecule, (**b**) canonical glycine and two DMSO molecules, (**c**) zwitterionic glycine and one DMSO molecule, and (**d**) zwitterionic glycine and two DMSO molecules calculated using B3LYP-D3BJ/6-311++G(d,p). Gray corresponds to carbons, blue to nitrogen, red to oxygen, and white to hydrogen. In the AIMAll molecular graphs, the small green, red, and blue spheres represent BCPs, RCPs, and CCPs, respectively. For the NCIPlot: Red surfaces correspond to repulsive forces, blue surfaces to moderate attractive forces, and green surfaces to weak attractive interactions. A contour value of 0.35 was used for the representation. Color code: Nitrogen, oxygen, sulfur, carbon, and hydrogen atoms are represented by blue, red, yellow, gray, and white spheres, respectively.

For the DMSO aggregates, the QTAIM analysis summarized in Tables S11 and S12 and Figure 13 shows that the Gly-DMSO cluster presents a bond critical point between the hydroxyl group of glycine and the O=S group of DMSO. The intramolecular distance, as shown in Figure 11, is 1.60 Å, consistent with a strong hydrogen bond. In fact, the bond between the O atom of the DMSO molecule and the H atom of the hydroxyl group of glycine presents a certain degree of covalency since the value of the total energy density is slightly negative and the |V(r)|/G(r) ratio is higher than 1. Additionally, two bond critical points between the carbonyl oxygen of glycine and one of the H atoms of the methyl groups of DMSO, with typical characteristics of hydrogen bonds, are also shown in good agreement with bond distances in Figure 11.

The NCI analysis confirms the attractive interactions seen with QTAIM. For the glycine–water complex (see Figure 12), we see how the hydroxyl group acts as a “strong” (in terms of non-covalent interactions) proton donor to the oxygen of water and how this, in turn, is a proton donor to the carbonyl group, with a somewhat weaker interaction, closing the cycle of interactions. There are also weak attractive interactions between the amino (-NH_2_) and the carbonyl (=O) groups, which are curiously more attractive than in the bare molecule, possibly due to an increase in the electron density of the carbonyl.

For the canonical glycine–DMSO complex (see Figure 13), graphically, we can see the electron density formed by the interactions mentioned with QTAIM. Like with a water molecule, the OH group of the carboxyl of glycine acts as a proton donor to the oxygen of the aggregated molecule (to the O=S group in this case, O-H•••O=S) through a considerably strong intermolecular interaction. In turn, now it is the methyl group that closes the ring through a hydrogen bond C-H•••O=C. As can be seen, this bond is somewhat weaker than the O-H•••O=S formed in the glycine–water pair, but, equally, it is considerably stabilizing.

In the case of zwitterionic glycine with DMSO, the NCI analysis indicates, in addition to the attractive interactions seen with QTAIM, that the NH_3_^+^ group acts as a strong proton donor in the hydrogen bond to the O=S group of a DMSO molecule and the O=C group of the same molecule. In addition, it is seen how the methyl groups of DMSO interact with the oxoanions (C=O^−^) of glycine. Despite being weak attractive interactions, they can be considered weak hydrogen bonds, and are important for stabilizing the cluster.

## 3. Discussion

We will start the discussion with observations from the explicit model. The results of this study provide valuable insights into the stabilization mechanisms of glycine in different solvent environments. Our findings demonstrate that DMSO interacts with glycine in ways that are fundamentally different from water, despite both being polar solvents. One of the most significant findings is that a single DMSO molecule can stabilize the zwitterionic form of glycine, while at least two water molecules are required for this purpose. This difference can be attributed to DMSO’s unique electronic properties and its ability to interact simultaneously with both charged moieties of the zwitterion: the S=O group of DMSO with the NH_3_^+^ group and the methyl groups of DMSO with the COO^−^ group.

Another observation is how the type I form of canonical glycine is the most stable configuration both for water and DMSO. For its complex with water, this allows a strong hydrogen bond between the hydroxyl group of glycine with the oxygen of water (O-H•••O), which is reinforced as more water molecules are added. This bond distance ranges from 1.7 to 1.8 Å for the hydrogen bonds involved. On the other hand, the bond is shorter for the hydrogen bond between the hydroxyl group of glycine with the oxygen of DMSO (O-H•••O=S), and the bond distance is around 1.6 Å. Additionally, the methyl groups take an active role in the stabilization of the clusters, with C-H•••O=C hydrogen bond distances ranging from 2.1 to 2.3 Å in both canonical and zwitterionic glycine clusters. Thus, we find the statement that “it is usually thought that DMSO can only function as an acceptor and that weak methyl hydrogen to electronegative intermolecular contacts probably do not constitute hydrogen bonds” [26,27,28,29,30] to be inappropriate, as our results suggest that the methyl groups do, in fact, participate in hydrogen bonding. Specifically, the QTAIM analysis reveals that all DMSO clusters exhibit critical points between the carbonyl oxygen of glycine and one of the hydrogen atoms of the methyl groups of DMSO. These interactions display characteristics typical of hydrogen bonds, such as low electron density, a positive Laplacian of electron density, a positive value of total energy density, and a |V(r)|/G(r) ratio less than 1. This can also be seen in the NCIplots, where the interactions range from weak attractive (green color) to moderate (green-bluish color).

In fact, the QTAIM analysis reveals that the interactions between DMSO and zwitterionic glycine have a partial covalent character, particularly in the bonds formed between the NH_3_^+^ hydrogens and the O atom of DMSO. This suggests a stronger interaction than typical hydrogen bonding, which contributes to the enhanced stabilization of the zwitterionic form.

Furthermore, and in relation to the above, another conclusion that we can draw at a general level is how DMSO interacts with any functional group, which could explain its application as a “universal solvent.” Not only is the S=O group key during this role, as chemical intuition indicates, but also the methyl groups. The results could explain why DMSO can interact with proteins through its solvation properties. DMSO is a great solvent, and it can surround protein molecules. In this work, we see the peculiarity of stabilizing both glycine in its neutral form and in the zwitterionic form, so it is not surprising that it can surround any amino acid (charged or not) of a protein. In addition, in this work, we see that DMSO creates aggregates efficiently both at a cooperative level between DMSO molecules and individually.

Additionally, another striking fact is how the water system prefers to self-aggregate regardless of whether it is the neutral or zwitterionic form of glycine, while the DMSO system does not have that preference. In the neutral form, it seems that DMSO is more stable when it self-aggregates, while the opposite occurs with glycine in its zwitterionic state. This non-preferential outcome of DMSO may explain why DMSO has proven to be one of the most versatile solvents due to its ability to penetrate and diffuse easily through the biological membranes.

Regarding the implicit solvent model, calculations provide a broader perspective on the behavior of glycine in bulk solvent environments. The temperature-dependent analysis reveals an interesting crossover point at approximately 150 K in DMSO, below which the zwitterionic form becomes more stable. This contrasts with aqueous environments where the zwitterionic form predominates at all biologically relevant temperatures. Nevertheless, the results also indicate that the relative free energy difference between canonical and zwitterionic glycine in DMSO is very small, and a solution would have an appreciable amount of the zwitterionic form in equilibrium with the canonical form at room temperature, particularly in water-DMSO solutions.

We believe that these findings have implications for protein crystallization protocols where DMSO is commonly used. The ability of DMSO to interact with both charged and neutral amino acid forms, combined with its temperature-dependent stabilization effects, suggests that careful control of DMSO concentration and temperature could be leveraged to influence protein conformation and crystal packing arrangements.

Finally, another point worth mentioning for all computational works or experimental work on cluster systems concerns molecular mechanics (MM) or other methodologies used for conformational searches. The case of glycine in DMSO is very illustrative. It is common that, due to computational resources, when hundreds of structures are obtained from a conformational search using molecular mechanics, researchers are limited to optimizing with Quantum Mechanics methods only the most stable structures (usually around 100 structures). Unfortunately, as we show, it is important to run all calculations. For certain systems, current force fields are not good enough to make a good estimation of the energies. As we show for glycine and two DMSO molecules, even using a 30 kJ mol^−1^ energy filtering and optimizing 627 structures, the global minimum was the structure at position #366 relative to the global minimum. This means that if we simply used the first one hundred structures provided by MM, we would have lost the 22 most stable structures below 8.06 kJ mol^−1^. For water, due to its strong aggregation power, this fact is not that drastic, but still structures such as #66 from glycine and two water molecules turned out to be relevant. Therefore, it is important to calculate as many structures as possible, or to make a conformational search using DFT methods or similar approaches.

In future studies, we plan on increasing the number of aggregated molecules. The trend in the explicit model suggests that additional DMSO molecules might eventually invert the stability order between the canonical and zwitterionic forms, so it would be interesting to add more DMSO molecules to the systems in future studies to test how many molecules are necessary to stabilize the zwitterionic form over the canonical form of glycine. Additionally, we plan to study binary mixtures between water and DMSO, as low concentrations of DMSO are usually employed during crystallization processes.

## 4. Materials and Methods

### 4.1. Computational Methods

All quantum chemical calculations were performed using the Gaussian16 software package [51]. The conformational searches were conducted using fast molecular mechanics methods using MMFFs [52] force field, which is designed to accurately represent the conformational energy of small organic molecules using empirical functions parameterized from experimental data and high-quality quantum calculations. This force field allows reliable estimation of the structures of different conformations without resorting to more expensive quantum-mechanical methods. During the search, multiple conformations were generated through rotations of single bonds and other internal degrees of freedom, followed by a local optimization of each structure according to the MMFFs parameters.

After the conformational search, all structures were optimized using the B3LYP functional [53,54,55] with the 6-311++G(d,p) [56] basis set, including empirical dispersion corrections (D3BJ) [57]. This level of theory provides a good balance between accuracy and computational cost for systems of this size and has been shown to reproduce experimental results for similar systems reliably. The B3LYP functional is a hybrid correlation-exchange functional that includes a correlation functional of Lee-Yang-Parr [14] and a hybrid exchange functional of Becke [15].

Frequency calculations were performed at the same level of theory to confirm that all optimized structures correspond to true minima on the potential energy surface (all real frequencies) and to obtain zero-point energy (ZPE) corrections and thermodynamic parameters. The RMS force convergence threshold has been set to 3 × 10^−4^ hartree/bohr, and the RMS displacement threshold to 1.2 × 10^−3^ bohr.

Solvent effects were modelled using both explicit molecules (1–2 molecules of water or DMSO) and implicit solvation via the integral equation formalism–polarizable continuum model (IEF-PCM) [58,59,60] and the solvation model based on electron density (SMD) [18]. These models represent a molecule within a cavity surrounded by a dielectric medium representing the solvent. The charge distribution of the solute (molecule) generates an electric polarization in the surrounding solvent, which is modelled as a homogeneous medium characterized by a permittivity. All calculations regarding both explicit and implicit solvation were performed at the B3LYP-D3BJ/6-311++G(d,p) level of theory.

The nature of the interactions between glycine and solvent molecules was analyzed using the AIMAll [47] and NCIPlot [48,49] programs.

### 4.2. Analysis of Non-Covalent Interactions

To characterize the nature of the interactions between glycine and solvent molecules, we employed two complementary approaches:

- Quantum Theory of Atoms in Molecules (QTAIM): This analysis was performed using the AIMAll program [47] to identify bond critical points (BCPs), ring critical points (RCPs) and cage critical points (CCPs) and determine the nature of the interactions based on the electron density (ρ), its Laplacian (∇^2^ρ), the total energy density (H), and the ratio between potential and kinetic energy densities (|V|/G). When ρ takes a high value and ∇^2^ρ < 0, the electronic charge is concentrated in the internuclear region, and it is said to be a shared interaction (characteristic of covalent bonds). In addition, in this type of interaction, the total energy density H(r) is negative and the ratio |V(r)|/G(r) > 2. When ρ is small and ∇^2^ρ > 0, we talk about closed-shell interaction, characteristic of ionic bonds, hydrogen bonds, or van der Waals molecules. In this type of interaction, the total energy density H(r) > 0 and the ratio |V(r)|/G(r) < 1.
- Non-covalent interaction (NCI) analysis: Using the NCIPlot program(3.0) [48,49] and VMD for visualization, we mapped the regions of non-covalent interactions in the molecular complexes, distinguishing between attractive interactions (hydrogen bonds), weak van der Waals forces, and repulsive interactions. The NCI analysis uses electron density to locate different types of intermolecular forces in the molecule. We can thus see the interactions that will stabilize or destabilize our conformer. We can distinguish 3 types of interactions: strong attractive interactions, such as hydrogen bonds (in blue color); strong repulsive interactions, such as steric repulsions (in red color); and van der Waals interactions, which are those weaker attractive interactions with lower electron density (in green color).

## 5. Conclusions

In this study, we have carried out a detailed computational investigation of glycine stabilization in both its canonical and zwitterionic forms when interacting with DMSO molecules, comparing these interactions with analogous water clusters. Our key findings are as follows:

- The zwitterionic form of glycine cannot be stabilized with a single water molecule using conventional quantum chemical levels such as B3LYP-D3BJ/6-311++G(d,p). In contrast, one DMSO molecule successfully stabilizes the zwitterionic form of glycine, demonstrating DMSO’s unique solvation properties. This stabilization occurs through specific interactions where the S=O group of DMSO acts as a hydrogen bond acceptor from the NH_3_^+^ group, while the methyl groups interact with the carboxylate group.
- QTAIM and NCI analyses reveal that some of these interactions have partial covalent character, particularly in the bonds formed between the NH_3_^+^ hydrogens and the O atom of DMSO, contributing to the enhanced stabilization of the zwitterionic form.
- In explicit solvation models, the canonical form of glycine remains more stable than the zwitterionic form with both water and DMSO molecules. However, two DMSO molecules significantly reduce this energy gap to approximately 12 kJ/mol, suggesting that additional DMSO molecules might eventually invert this stability.
- In implicit solvent models, the zwitterionic form becomes more stable than the canonical form in both water and DMSO at 0 K. However, temperature-dependent analysis reveals that in DMSO, the canonical form becomes more stable above 150 K, while in water, the zwitterionic form predominates up to about 375 K.
- DMSO exhibits remarkable versatility in forming hydrogen bonds, not only through its S=O group acting as a hydrogen bond acceptor but also through its methyl groups forming C-H•••O interactions with carbonyl groups of glycine.
- The self-aggregation patterns differ significantly between water and DMSO clusters: water molecules strongly interact with each other regardless of whether glycine is in its canonical or zwitterionic form, while DMSO shows different preferences depending on the glycine form.

These findings contribute to resolving some of the contradictory reports in the literature regarding the minimum requirements for zwitterion stabilization and provide molecular-level insights into the role of DMSO in biomolecular stabilization and crystallization. The unique ability of DMSO to stabilize both canonical and zwitterionic forms of glycine, combined with its temperature-dependent stabilization effects, suggests that careful control of DMSO concentration and temperature could be leveraged to influence protein conformation and crystal packing arrangements in crystallization protocols.

## Figures and Tables

**Table 1:** Number of conformers generated via molecular mechanics.

System	Canonical	Zwitterionic
Gly	9	3
Gly—H_2_O	47	4
Gly—2 H_2_O	71	13
Gly—DMSO	110	4
Gly—2 DMSO	616	45

**Table 2:** Relative energies between the most stable structure of canonical glycine and its zwitterionic form in water and in DMSO using B3LYP-D3BJ/6-311++G(d,p). “Can.” indicates that glycine is in its canonical state, while “Zwitt.” indicates that glycine is in its zwitterionic state.

	H_2_O		2H_2_O		DMSO		2DMSO	
Parameter	Can.	Zwitt.	Can.	Zwitt.	Can.	Zwitt.	Can.	Zwitt.
ΔE ^1^	-	-	0	45.15	-	40.89	0	7.09
ΔE_ZPE_ ^2^	-	-	0	46.57	-	43.74	0	12.13
ΔG ^3^	-	-	0	49.19	-	48.98	0	19.06

^1^ Relative energies (in kJ mol^−1^) with respect to the global minimum. ^2^ Relative energies (in kJ mol^−1^) with respect to the global minimum, considering the zero-point energy (ZPE). ^3^ Relative Gibbs energies (in kJ mol^−1^) calculated at 298 K and 1 atm.

**Table 3:** Relative energies between the most stable structure of canonical glycine and its zwitterionic form in water and DMSO using a polarizable continuum model (PCM) and the solvation model based on electron density (SMD) at 310 K and 1 atm.

	H_2_O		DMSO	
Parameter	Canonical	Zwitterionic	Canonical	Zwitterionic
ΔE ^1^	6.09/27.25	0/0	4.83/9.78	0/0
ΔE_ZPE_ ^2^	1.47/22.30	0/0	0.25/5.43	0/0
ΔG ^3^	0.39/22.99	0/0	0/4.64	0.90/0.00

^1^ Relative energies (in kJ mol^−1^) with respect to the global minimum. ^2^ Relative energies (PCM/SMD in kJ mol^−1^) with respect to the global minimum, considering the zero-point energy (ZPE). ^3^ Relative Gibbs energies (in kJ mol^−1^) calculated at 298 K and 1 atm. The calculations were performed at the B3LYP-D3BJ/6-311++G(d,p) level.

## Data Availability

The original contributions presented in this study are included in the article/Supplementary Materials. Further inquiries can be directed to the corresponding authors.

## Supplementary Materials

The following supporting information can be downloaded at: https://www.mdpi.com/article/10.3390/ph18081168/s1. Figure S1: Most stable structures of canonical glycine in isolated conditions; Figure S2: Relaxed PES of 3-CInp-7 calculated at B3LYP-D3BJ/6-311++G(d,p); Table S1: Theoretical spectroscopic parameters for the calculated structures of glycine at B3LYP-D3BJ/6-311++G(d,p); Figure S3: Most stable structures of canonical glycine with one water molecule in isolated conditions; Figure S4: Relaxed PES of 3-CInp-W1-12 calculated at B3LYP-D3BJ/6-311++G(d,p); Table S2: title; Theoretical spectroscopic parameters for the calculated structures of glycine-H2O at B3LYP-D3BJ/6-311++G(d,p); Figure S5: Most stable structures of canonical glycine with two water molecules in isolated conditions; Table S3: Theoretical spectroscopic parameters for the calculated structures of glycine-2H2O at B3LYP-D3BJ/6-311++G(d,p); Figure S6: Most stable structures of zwitterionic glycine with two water molecules in isolated conditions; Table S4: Theoretical spectroscopic parameters for the calculated structures of zwitterionic glycine and two water molecules at B3LYP-D3BJ/6-311++G(d,p); Figure S7: Most stable structures of canonical glycine with one DMSO molecule in isolated conditions; Table S5: Theoretical spectroscopic parameters for the calculated structures of glycine–DMSO at B3LYP-D3BJ/6-311++G(d,p); Figure S8: Most stable structures of canonical glycine with two DMSO molecule in isolated conditions; Table S6: Theoretical spectroscopic parameters for the calculated structures of glycine-2DMSO at B3LYP-D3BJ/6-311++G(d,p); Figure S9: Most stable structures of zwitterionic glycine with one DMSO molecule in isolated conditions; Table S7: Theoretical spectroscopic parameters for the calculated structures of zwitterionic glycine and one DMSO molecule at B3LYP-D3BJ/6-311++G(d,p); Figure S10: Most stable structures of zwitterionic glycine with two DMSO molecules in isolated conditions; Table S8: Theoretical spectroscopic parameters for the calculated structures of zwitterionic glycine and two DMSO molecules at B3LYP-D3BJ/6-311++G(d,p); Table S9: Topological properties at the bond critical points (BCP) for the most stable glycine conformer calculated using the QTAIM analysis at B3LYP/6-311++G(d,p); Table S10: Topological properties at the bond critical points (BCP) for the most stable conformer of glycine with one and two water molecules and the zwitterionic form of glycine with one water molecule calculated using the QTAIM analysis at B3LYP/6-311++G(d,p). Table S11: Topological properties at the bond critical points (BCP) for the most stable conformer of glycine and its zwitterionic form with one DMSO molecule calculated using the QTAIM analysis at B3LYP/6-311++G(d,p). Table S12: Topological properties at the bond critical points (BCP) for the most stable conformer of glycine and its zwitterionic form with two DMSO molecules calculated using the QTAIM analysis at B3LYP/6-311++G(d,p). XYZ files for all the structures.

## Abbreviations

The following abbreviations are used in this manuscript:

B3LYP	Becke Three-Parameter Hybrid Functional
BCP	Bond critical point
CCP	Cage critical point
HF	Hartree–Fock
NCI	Non-covalent interactions
PES	Potential energy surface
QTAIM	Quantum Theory of Atoms In Molecules
RCP	Ring critical point
RGB	Red green blue
MM	Molecular mechanics
DMSO	Dimethyl sulfoxide
ZPE	Zero-point energy
MMFFs	Merck Molecular Force Field Static
PCM	Polarizable continuum model
SMD	Solvation model based on electron density
Gly	Glycine

## References

[1] Rand R.P., Ho M.W., Littlechild J.A., Finney J.L., Cupane A., Engberts J.B.F.N. (2004). Probing the role of water in protein conformation and function. Philos. Trans. R. Soc. B Biol. Sci..

[2] Timasheff S.N. (1970). Protein-Solvent Interactions and Protein Conformation. Acc. Chem. Res..

[3] Levy Y., Onuchic J.N. (2006). Water Mediation In Protein Folding And Molecular Recognition. Annu. Rev. Biophys. Biomol. Struct..

[4] Canchi D.R., García A.E. (2013). Cosolvent effects on protein stability. Annu. Rev. Phys. Chem..

[5] Panuszko A., Pastwa P., Gajewski J., Bruździak P. (2024). Characterizing Interactions Between Small Peptides and Dimethyl Sulfoxide Using Infrared Spectroscopy and Computational Methods. Molecules.

[6] Levy H.A., Corey R.B. (1941). The crystal structure of dl-alanine. J. Am. Chem. Soc..

[7] Donohue J. (1950). The Crystal Structure of dl-Alanine. II. Revision of Parameters by Three-Dimensional Fourier Analysis. J. Am. Chem. Soc..

[8] Albrecht G., Corey R.B. (1939). The Crystal Structure of Glycine. J. Am. Chem. Soc..

[9] Marsh R.E., Donohue J. (1967). Crystal Structure Studies of Amino Acids and Peptides. Adv. Protein Chem..

[10] Kieffer W.F. (1972). Dimethyl sulfoxide. Vol.1: Basic concepts of DMSO (Jacob, Stanley W.; Rosenbaum, Edward E.; Wood, Don C.). J. Chem. Educ..

[11] Huang A., Liu C., Ma L., Tong Z., Lin R. (2012). Effects of clustering structure on volumetric properties of amino acids in (DMSO + water) mixtures. J. Chem. Thermodyn..

[12] Arakawa T., Kita Y., Timasheff S.N. (2007). Protein precipitation and denaturation by dimethyl sulfoxide. Biophys. Chem..

[13] Voets I.K., Cruz W.A., Moitzi C., Lindner P., Arêas E.P.G., Schurtenberger P. (2010). DMSO-induced denaturation of hen egg white lysozyme. J. Phys. Chem. B.

[14] Tjernberg A., Markova N., Griffiths W.J., Hallén D. (2006). DMSO-related effects in protein characterization. J. Biomol. Screen..

[15] Chaudhari A., Lee S.L. (2005). Computational study of glycine-(water)3 complex by density functional method. Chem. Phys..

[16] Vener M.V., Medvedev A.G., Churakov A.V., Prikhodchenko P.V., Tripol’Skaya T.A., Lev O. (2011). H-bond network in amino acid cocrystals with H_2_O or H_2_O_2_. the DFT study of serine—H_2_O and Serine—H_2_O_2_. J. Phys. Chem. A.

[17] Kohn W., Sham L.J. (1965). Self-consistent equations including exchange and correlation effects. Phys. Rev..

[18] Marenich A.V., Cramer C.J., Truhlar D.G. (2009). Universal solvation model based on solute electron density and on a continuum model of the solvent defined by the bulk dielectric constant and atomic surface tensions. J. Phys. Chem. B.

[19] Yang G., Zhou L., Chen Y. (2016). Stabilization of zwitterionic versus canonical proline by water molecules. Springerplus.

[20] Kim J.Y., Ahn D.S., Park S.W., Lee S. (2014). Gas phase hydration of amino acids and dipeptides: Effects on the relative stability of zwitterion vs. canonical conformers. RSC Adv..

[21] Ulman K., Busch S., Hassanali A.A. (2018). Quantum mechanical effects in zwitterionic amino acids: The case of proline, hydroxyproline, and alanine in water. J. Chem. Phys..

[22] Kokpol S.U., Doungdee P.B., Hannongbua S.V., Rode B.M., Limtrakul J.P. (1988). Ab initio study of the hydration of the glycine zwitterion. J. Chem. Soc. Faraday Trans. 2 Mol. Chem. Phys..

[23] Ding Y., Krogh-Jespersen K. (1996). The 1: 1 glycine zwitterion-water complex: An ab initio electronic structure study. J. Comput. Chem..

[24] Langlet J., Caillet J., Evleth E., Kassab E., Rivail J.L. (1990). Studies in Physical and Theoretical Chemistry.

[25] Wanga W., Pu X., Zhenga W., Wong N.B., Tian A. (2003). Some theoretical observations on the 1:1 glycine zwitterion-water complex. J. Mol. Struct. THEOCHEM.

[26] Luzar A., Soper A.K., Chandler D. (1993). Combined neutron diffraction and computer simulation study of liquid dimethyl sulphoxide. J. Chem. Phys..

[27] McLain S.E., Soper A.K., Luzar A. (2007). Investigations on the structure of dimethyl sulfoxide and acetone in aqueous solution. J. Chem. Phys..

[28] Soper A.K. (2012). Computer simulation as a tool for the interpretation of total scattering data from glasses and liquids. Mol. Simul..

[29] Onthong U., Megyes T., Bakó I., Radnai T., Grósz T., Hermansson K., Probst M. (2004). X-ray and neutron diffraction studies and molecular dynamics simulations of liquid DMSO. Phys. Chem. Chem. Phys..

[30] Di Mino C., Clancy A.J., Sella A., Howard C.A., Headen T.F., Seel A.G., Skipper N.T. (2023). Weak Interactions in Dimethyl Sulfoxide (DMSO)-Tertiary Amide Solutions: The Versatility of DMSO as a Solvent. J. Phys. Chem. B.

[31] Halgren T.A. (1999). MMFF VI. MMFF94s option for energy minimization studies. J. Comput. Chem..

[32] (2018). Schrödinger Release 2018-3.

[33] Hu C.H., Shen M., Schaefer H.F. (1993). Glycine Conformational Analysis. J. Am. Chem. Soc..

[34] Ke H.W., Rao L., Xu X., Yan Y.J. (2008). Theoretical study of glycine conformers. J. Theor. Comput. Chem..

[35] Barone V., Biczysko M., Bloino J., Puzzarini C. (2013). Glycine conformers: A never-ending story?. Phys. Chem. Chem. Phys..

[36] Lovas F.J., Kawashima Y., Grabow J.-U., Suenram R.D., Fraser G.T., Hirota E. (1995). Microwave spectra, hyperfine structure, and electric dipole moments for conformers I and II of glycine. Astrophys. J..

[37] Schafer L., Sellers H.L., Lovas F.J., Suenram R.D. (1980). Theory versus Experiment: The Case of Glycine. J. Am. Chem. Soc..

[38] Suenram R.D., Lovas F.J. (1980). Millimeter Wave Spectrum of Glycine. A New Conformer. J. Am. Chem. Soc..

[39] Stepanian S.G., Reva I.D., Radchenko E.D., Rosado M.T.S., Duarte M.L.T.S., Fausto R., Adamowicz L. (1998). Matrix-isolation infrared and theoretical studies of the glycine conformers. J. Phys. Chem. A.

[40] Ruoff R.S., Klots T.D., Emilsson T., Gutowsky H.S. (1990). Relaxation of conformers and isomers in seeded supersonic jets of inert gases. J. Chem. Phys..

[41] Godfrey P.D., Brown R.D., Rodgers F.M. (1996). The missing conformers of glycine and alanine: Relaxation in seeded supersonic jets. J. Mol. Struct..

[42] Alonso J.L., Cocinero E.J., Lesarri A., Sanz M.E., López J.C. (2006). The glycine-water complex. Angew. Chemie—Int. Ed..

[43] Balabin R.M. (2010). The first step in glycine solvation: The glycine-water complex. J. Phys. Chem. B.

[44] Brown G.G., Dian B.C., Douglass K.O., Geyer S.M., Shipman S.T., Pate B.H. (2008). A broadband Fourier transform microwave spectrometer based on chirped pulse excitation. Rev. Sci. Instrum..

[45] Alonso J.L., López J.C. (2014). Microwave Spectroscopy of Biomolecular Building Blocks. Top. Curr. Chem..

[46] Alonso J.L., Peña I., Sanz M.E., Vaquero V., Mata S., Cabezas C., López J.C. (2013). Observation of dihydrated glycine. Chem. Commun..

[47] Keith T.A. (2019). AIMAll.

[48] Contreras-García J., Johnson E.R., Keinan S., Chaudret R., Piquemal J.P., Beratan D.N., Yang W. (2011). NCIPLOT: A program for plotting noncovalent interaction regions. J. Chem. Theory Comput..

[49] Johnson E.R., Keinan S., Mori-Sánchez P., Contreras-García J., Cohen A.J., Yang W. (2010). Revealing noncovalent interactions. J. Am. Chem. Soc..

[50] Humphrey W., Dalke A., Schulten K. (1996). VMD: Visual molecular dynamics. J. Mol. Graph..

[51] Frisch M.J., Trucks G.W., Schlegel H.B., Scuseria G.E., Robb M.A., Cheeseman J.R., Scalmani G., Barone V., Mennucci B., Petersson G.A. (2012). Gaussian.

[52] Halgren T.A. (1996). Merck molecular force field. I. Basis, form, scope, parameterization, and performance of MMFF94. J. Comput. Chem..

[53] Becke A.D. (1988). Density-functional exchange-energy approximation with correct asymptotic behavior. Phys. Rev. A.

[54] Becke A.D. (1993). A new mixing of Hartree–Fock and local density-functional theories. J. Chem. Phys..

[55] Zhao Y., Truhlar D.G. (2007). Density functionals for noncovalent interaction energies of biological importance. J. Chem. Theory Comput..

[56] Frisch M.J., Pople J.A., Binkley J.S. (1984). Self-consistent molecular orbital methods 25. Supplementary functions for Gaussian basis sets. J. Chem. Phys..

[57] Schwabe T., Grimme S. (2007). Double-hybrid density functionals with long-range dispersion corrections: Higher accuracy and extended applicability. Phys. Chem. Chem. Phys..

[58] Mennucci B., Tomasi J. (1997). Continuum solvation models: A new approach to the problem of solute’s charge distribution and cavity boundaries. J. Chem. Phys..

[59] Cancès E., Mennucci B., Tomasi J. (1997). A new integral equation formalism for the polarizable continuum model: Theoretical background and applications to Isotropic and anisotropic dielectrics. J. Chem. Phys..

[60] Cossi M., Barone V., Mennucci B., Tomasi J. (1998). Ab initio study of ionic solutions by a polarizable continuum dielectric model. Chem. Phys. Lett..

