# Supplementary material for: Stabilization of Zwitterionic Versus Canonical Glycine by DMSO Molecules
Source: Pharmaceuticals (Basel). 2025 Aug 6;18(8):1168. doi: 10.3390/ph18081168 (PMC12389192; doi:10.3390/ph18081168)
Supplement: Supplementary file 1 [file pharmaceuticals-18-01168-s001.zip › pharmaceuticals-Manuscript-SI.pdf]

Supporting information for:

Stabilization of Zwitterionic versus Canonical Glycine by DMSO Molecules

Figure S1. Most stable structures of canonical glycine in isolated conditions. Relative electronic energies considering the zero-point energy and relative Gibbs free energies at room temperature calculated using B3LYP-D3BJ/6-311++G(d,p) are also indicated ($\Delta E_{ZPE}/\Delta G$). Values are given in kJ/mol.

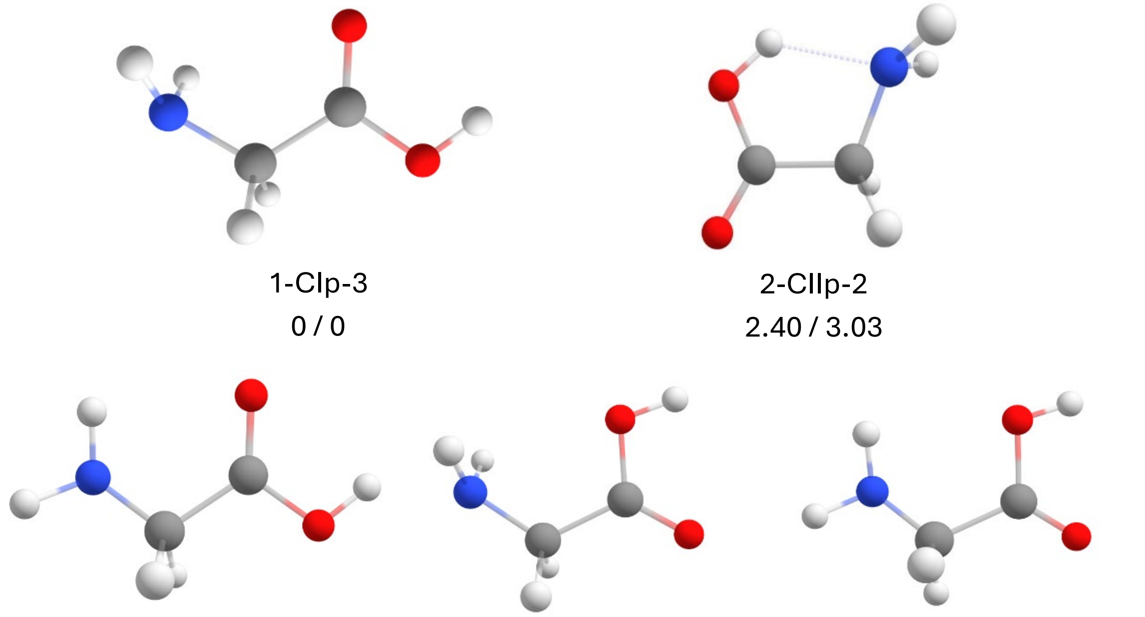

Figure S2. Relaxed PES of 3-CInp-7 calculated at B3LYP-D3BJ/6-311++G(d,p) rotating the C-C-N-H dihedral angle. As can be seen the interconversion barrier height from conformer 3-CInp-7 to 1-CIp-3 is about 0.72 kJ mol^{-1}.

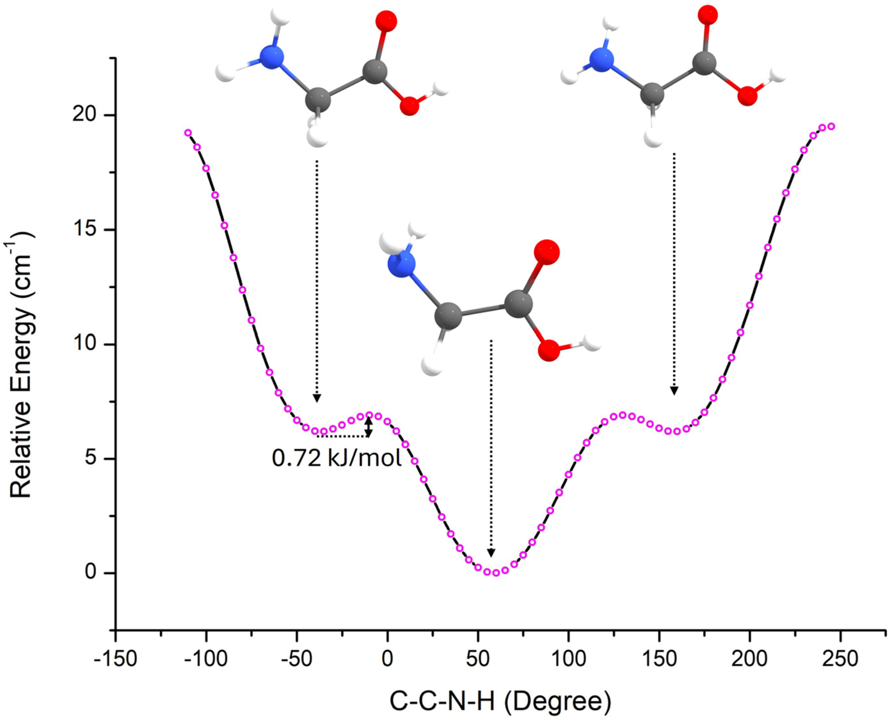

Table S1. Theoretical spectroscopic parameters for the calculated structures of Glycine at B3LYP-D3BJ/6-311++G(d,p).

Parameters	1-CIp-3	2-CIIp-2	3-CInp-7	4-CIIIp-9	5-CIIInp-6
**A[a]**	10345	10161	10404	9982	9799
**B**	3849	4062	3952	3967	4046
**C**	2897	3007	2948	2933	3020
**μ_a**	-1.0	-5.7	0.2	0.5	-1.6
**μ_b**	-0.6	-0.9	-1.6	-1.8	1.0
**μ_c**	0.0	0.0	1.4	0.0	1.6
**χ_{aa}**	-1.427	1.9014	2.9944	-1.7911	2.8101
**χ_{aa}**	-0.6335	-3.7414	2.4099	-0.3289	2.3826
**χ_{aa}**	2.0605	1.8401	-5.4044	2.12	-5.1927
**ΔE[b]**	0	1.02	6.18	6.33	11.31
**ΔE_{ZPE}[c]**	0	2.38	5.85	6.44	11.34
**ΔG[d]**	0	2.84	6.55	3.48	11.68

[a] *A*, *B* and *C* represent the rotation constants (in MHz); μ_a, μ_b and μ_c are the components of the electric dipole moment (in D). χ_{aa}, χ_{bb} y χ_{cc} are the diagonal elements of the ^{14}N nuclear quadrupole coupling tensor in MHz. [b]Relative energies (in kJ mol^{-1}) with respect to the global minimum. [c]Relative energies (in kJ mol^{-1}) with respect to the global minimum, considering the zero-point energy (ZPE). [d]Gibbs energies (in kJ mol^{-1}) calculated at 298 K and 1 atm.

Figure S3. Most stable structures of canonical glycine with one water molecule in isolated conditions. Relative electronic energies considering the zero-point energy and relative Gibbs free energies at room temperature calculated using B3LYP-D3BJ/6-311++G(d,p) are also indicated ($\Delta E_{ZPE}/\Delta G$). Values are given in kJ/mol.

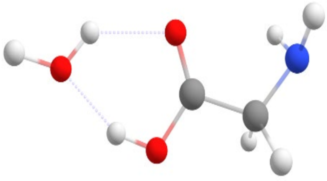

1-CIp-1W-1
0/0

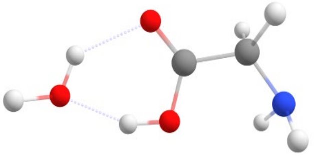

2-CIIIp-1W-4
4.93/3.81

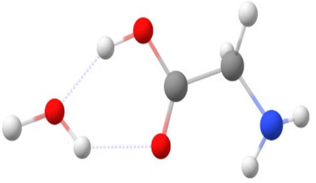

3-CInp-1W-12
5.09/6.04

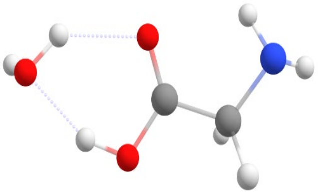

4-CInp-1W-44
5.53/6.31

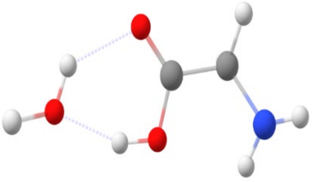

5-CIIInp-1W-13
9.98/10.68

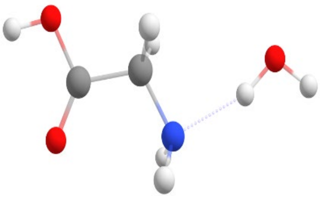

6-CIp-1W-33
13.23/6.59

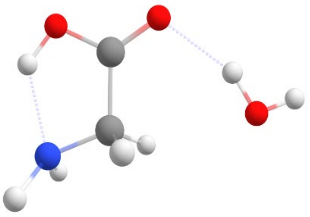

7-CIIp-1W-6
13.27/8.86

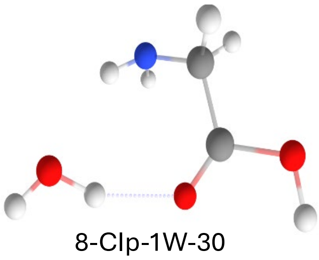

8-CIp-1W-30
14.38/12.55

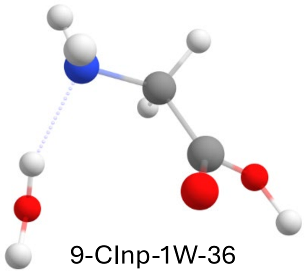

9-CInp-1W-36
16.85/12.71

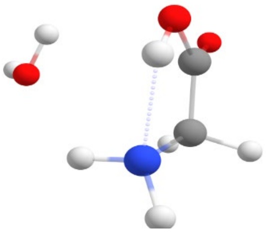

10-CIIp-1W-15
17.23/15.84

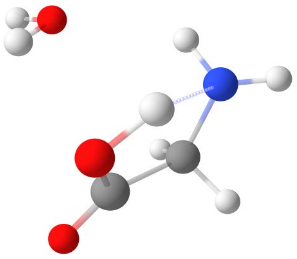

11-CIIp-1W-3
17.25/15.90

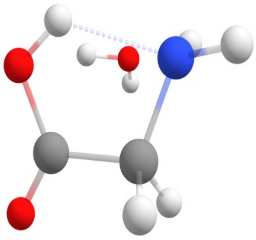

12-CIIp-1W-16
17.31/15.76

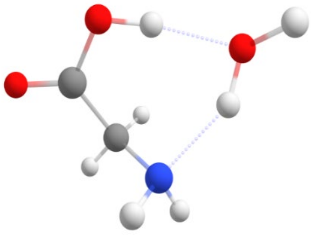

13-CIV-1W-20
18.44/20.94

Figure S3. Continuation. Most stable structures of canonical glycine with one water molecule in isolated conditions. Relative electronic energies considering the zero-point energy and relative Gibbs free energies at room temperature calculated using B3LYP-D3BJ/6-311++G(d,p) are also indicated ($\Delta E_{ZPE}/\Delta G$). Values are given in kJ/mol.

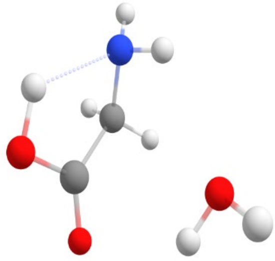

14-CIIp-1W-17
18.47/16.84

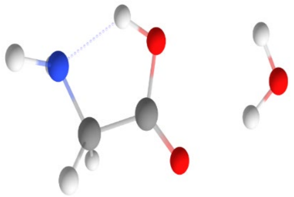

15-CIIp-1W-9
19.93/9.60

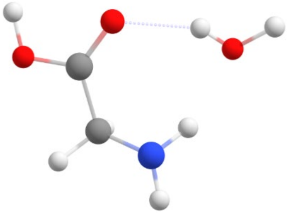

16-CInp-1W-42
19.94/18.99

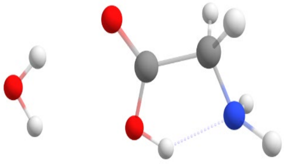

17-CIIp-1W-5
19.96/10.37

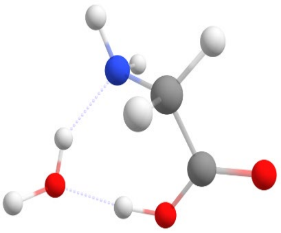

18-CIV-1W-23
20.46/22.52

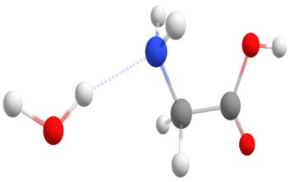

19-CIIIp-1W-39
20.81/8.25

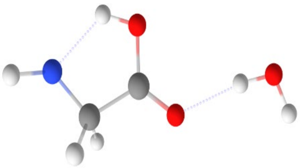

20-CIIp-1W-11
21.16/11.36

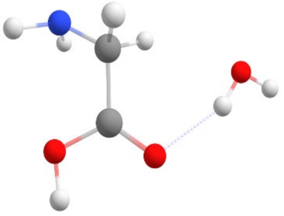

21-CIIIp-1W-47
24.35/18.91

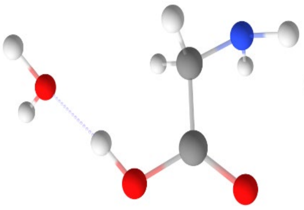

22-CIp-1W-24
28.73/22.96

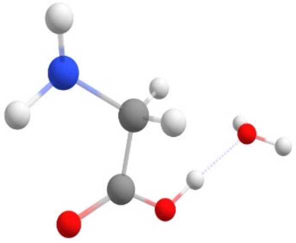

23-CInp-1W-46
34.09/28.53

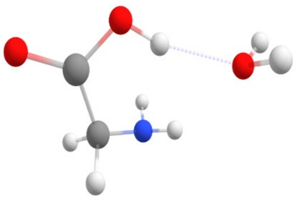

24-CIV-1W-29
36.01/31.98

Figure S4. Relaxed PES of 3-CInp-W1-12 calculated at B3LYP-D3BJ/6-311++G(d,p) rotating the C-C-N-H dihedral angle. As can be seen the interconversion barrier height from conformer 3-CInp-W1-12 to 1-CIp-W1-1 is about 0.83 kJ mol^{-1}.

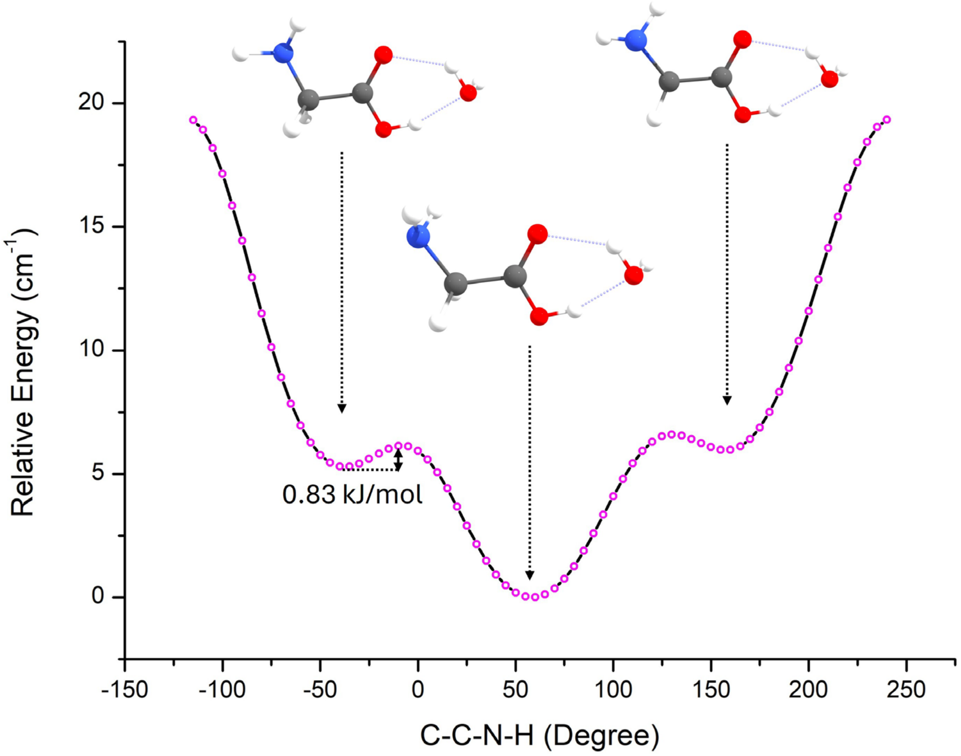

Table S2. Theoretical spectroscopic parameters for the calculated structures of Glycine-H_2O at B3LYP-D3BJ/6-311++G(d,p).

Par.	1-CIp-1W-1	2-CIIIp-1W-4	3-CInp-1W-12	4-CInp-1W-44	5-CIIInp-1W-13	6-CIp-1W-33	7-CIIp-1W-6	8-CIp-1W-30	9-CInp-1W-36	10-CIIp-1W-15
A[a]	8449	8094	8461	8455	8055	7416	4517	4329	3898	3450
B	1631	1668	1660	1659	1687	1317	2051	2162	2136	2669
C	1392	1408	1411	1411	1435	1133	1439	1559	1831	2019
μ_a	1.2	-1.1	-0.1	0.2	0.5	-1.4	-2.1	1.4	2.3	-3.1
μ_b	-0.5	-0.7	0.1	-0.2	-0.4	1.1	-3.5	-1.7	2.5	2.2
μ_c	1.2	1.2	0.1	2.6	0.1	0.0	-0.1	0.9	0.3	0.9
χ_{aa}	-3.7781	-4.1763	2.5996	2.5981	2.3869	-4.268	0.1322	2.845	-0.1972	2.1994
χ_{aa}	1.7054	2.0711	2.7828	2.7958	2.773	2.3968	-2.0736	-4.6446	-2.0496	1.7383
χ_{aa}	2.0727	2.1052	-5.3824	-5.3939	-5.1599	1.8712	1.9414	1.7996	2.2468	-3.9377
ΔE[b]	0	4.68	5.29	5.97	9.84	14.71	14.10	15.75	17.92	18.27
ΔE_{ZPE}[c]	0	4.94	5.10	5.54	9.98	13.23	13.28	14.38	16.85	17.24
ΔG[d]	0	3.84	6.06	6.33	10.70	6.61	8.89	12.58	12.74	15.86

[a] *A*, *B* and *C* represent the rotation constants (in MHz); μ_a, μ_b and μ_c are the components of the electric dipole moment (in D). χ_{aa}, χ_{bb} y χ_{cc} are the diagonal elements of the ^{14}N nuclear quadrupole coupling tensor in MHz. [b]Relative energies (in kJ mol^{-1}) with respect to the global minimum. [c]Relative energies (in kJ mol^{-1}) with respect to the global minimum, considering the zero-point energy (ZPE).[d]Gibbs energies (in kJ mol^{-1}) calculated at 298 K and 1 atm.

Table S2. Continuation. Theoretical spectroscopic parameters for the calculated structures of Glycine-H_2O at B3LYP-D3BJ/6-311++G(d,p).

Par.	11-CIIp-1W-3	12-CIIp-1W-16	13-CIV-1W-20	14-CIIp-1W-17	15-CIIp-1W-9	16-CInp-1W-42	17-CIIp-1W-5	18-CIV-1W-23	19-CIIIp-1W-39	20-CIIp-1W-11
A[a]	3458	3450	4756	3143	8487	4528	8484	4776	7539	9910
B	2664	2671	2315	2976	1464	2124	1464	2267	1314	1257
C	2017	2024	1673	2075	1267	1529	1267	1633	1134	1131
μ_a	-3.1	3.1	4.4	-2.2	-8.7	-1.3	-8.7	-4.2	-1.1	-7.7
μ_b	2.2	2.2	3.8	3.1	1.1	0.1	-1.1	4.6	4.0	-2.2
μ_c	-0.9	0.9	-0.8	0.0	0.0	0.3	0.0	1.5	0.0	-0.1
χ_{aa}	2.2056	2.194	-0.923	-0.5216	-0.3556	2.6264	-0.3495	-0.9517	-4.3944	1.322
χ_{aa}	1.729	1.7439	-1.3504	2.5233	-1.5532	2.1534	-1.5514	-1.0882	2.4634	-3.1972
χ_{aa}	-3.9346	-3.9379	2.2734	-2.0017	1.9088	-4.7798	1.9008	2.0399	1.931	1.8752
ΔE[b]	18.27	18.29	15.80	19.17	22.14	21.03	22.14	18.63	22.33	23.65
ΔE_{ZPE}[c]	17.26	17.31	18.45	18.48	19.93	19.95	19.96	20.47	20.81	21.16
ΔG[d]	15.92	15.78	20.96	16.86	9.62	19.01	10.40	22.54	8.27	11.39

[a] *A*, *B* and *C* represent the rotation constants (in MHz); μ_a, μ_b and μ_c are the components of the electric dipole moment (in D). χ_{aa}, χ_{bb} y χ_{cc} are the diagonal elements of the ^{14}N nuclear quadrupole coupling tensor in MHz. [b]Relative energies (in kJ mol^{-1}) with respect to the global minimum. [c]Relative energies (in kJ mol^{-1}) with respect to the global minimum, considering the zero-point energy (ZPE).[d]Gibbs energies (in kJ mol^{-1}) calculated at 298 K and 1 atm.

Table S2. Continuation. Theoretical spectroscopic parameters for the calculated structures of Glycine-H_2O at B3LYP-D3BJ/6-311++G(d,p).

Parameters	21-CIIIp-1W-47	22-CIp-1W-24	23-CInp-1W-46	24-CIV-1W-29
A^a	4237	4409	4566	4498
B	2019	1886	1862	2007
C	1404	1350	1352	1458
μ_a	1.7	-5.4	5.6	-6.3
μ_b	2.3	-2.7	-3.4	0.2
μ_c	-0.7	-0.2	1.4	0.0
χ_{aa}	2.8652	3.0089	3.1291	2.8664
χ_{aa}	-4.5991	-4.8907	1.7824	-4.8544
χ_{aa}	1.7339	1.8818	-4.9114	1.988
ΔE^b	26.88	32.30	37.94	38.84
$\Delta E_{ZPE}{}^c$	24.36	28.74	34.09	36.01
ΔG^d	18.93	22.98	28.55	32.00

[a] *A*, *B* and *C* represent the rotation constants (in MHz); μ_a, μ_b and μ_c are the components of the electric dipole moment (in D). χ_{aa}, χ_{bb} y χ_{cc} are the diagonal elements of the ^{14}N nuclear quadrupole coupling tensor in MHz. [b]Relative energies (in kJ mol^{-1}) with respect to the global minimum. [c]Relative energies (in kJ mol^{-1}) with respect to the global minimum, considering the zero-point energy (ZPE).[d]Gibbs energies (in kJ mol^{-1}) calculated at 298 K and 1 atm.

Figure S5. Most stable structures of canonical glycine with two water molecules in isolated conditions. Relative electronic energies considering the zero-point energy and relative Gibbs free energies at room temperature calculated using B3LYP-D3BJ/6-311++G(d,p) are also indicated (ΔE_{ZPE}/ΔG). Values are given in kJ/mol.

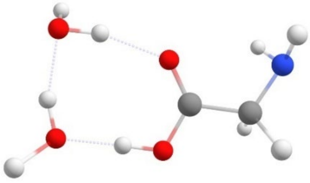

1-CIp-2W-6
0/0

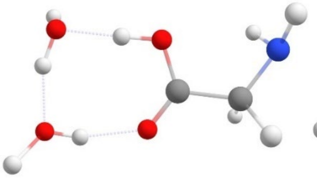

2-CIIIp-2W-66
4.25/3.45

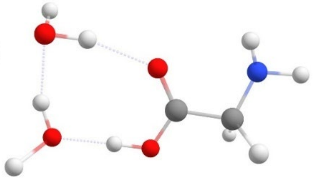

3-CInp-2W-9
4.99/5.99

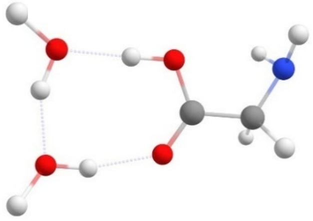

4-CIIIp-2W-7
5.87/4.06

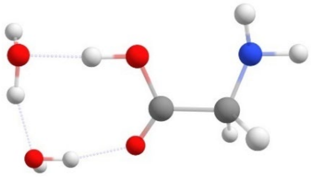

5-CIIInp-2W-10
10.89/11.03

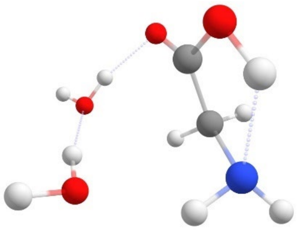

6-CIIp-2W-28
16.45/16.83

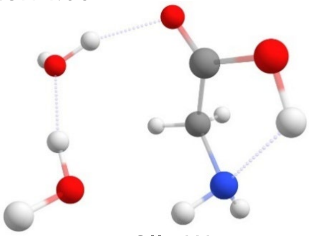

7-CII-2W-19
16.47/15.94

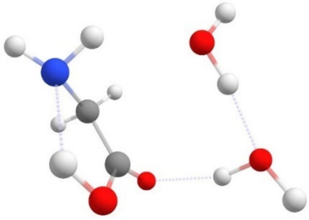

8-CIIp-2W-15
17.27/16.81

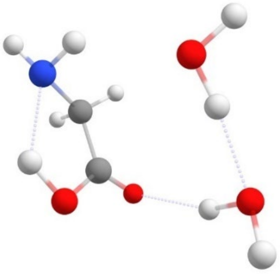

9-CIIp-2W-4
17.29/16.89

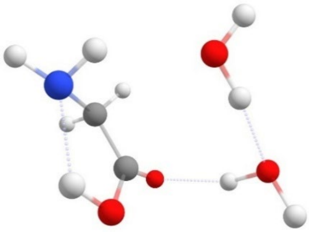

10-CIIp-2W-12
17.31/16.98

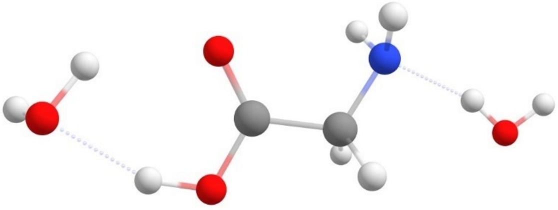

11-CIp-2W-30
20.02/13.49

Figure S5. Continuation. Most stable structures of canonical glycine with two water molecules in isolated conditions. Relative electronic energies considering the zero-point energy and relative Gibbs free energies at room temperature calculated using B3LYP-D3BJ/6-311++G(d,p) are also indicated ($\Delta E_{ZPE}/\Delta G$). Values are given in kJ/mol.

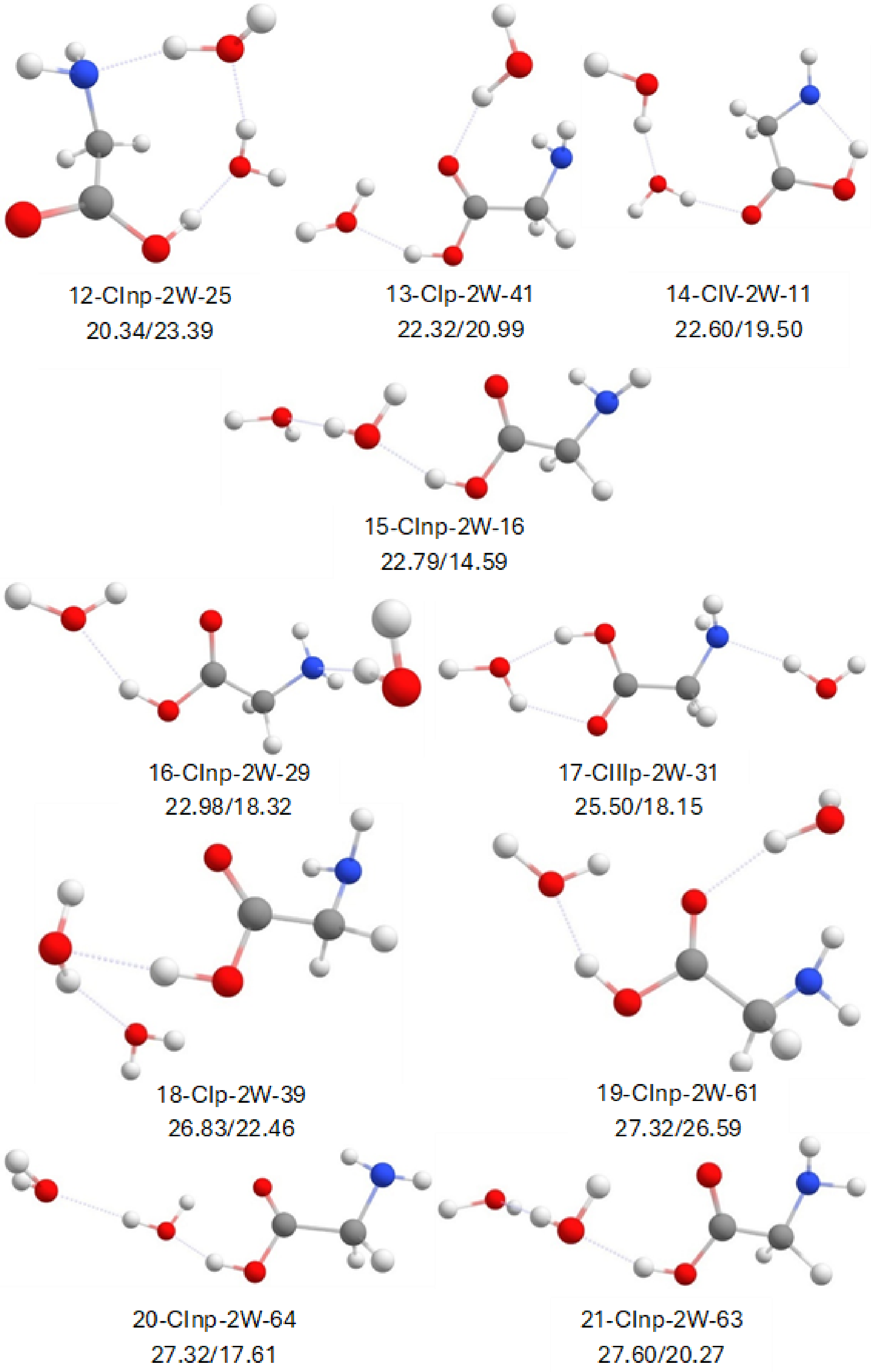

Figure S5. Continuation. Most stable structures of canonical glycine with two water molecules in isolated conditions. Relative electronic energies considering the zero-point energy and relative Gibbs free energies at room temperature calculated using B3LYP-D3BJ/6-311++G(d,p) are also indicated (ΔE_{ZPE}/ΔG). Values are given in kJ/mol.

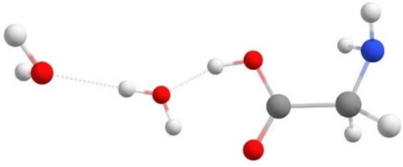

22-CIIIp-2W-21
27.79/18.15

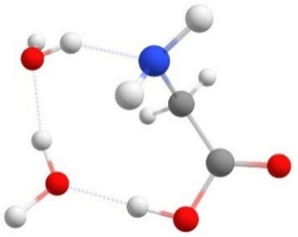

23-CIV-2W-14
29.38/30.71

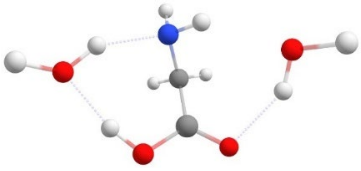

24-CIV-2W-35
29.52/32.06

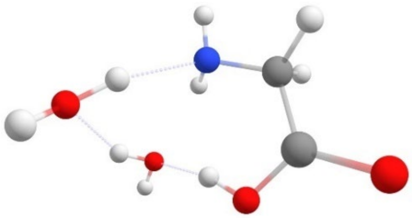

25-CIV-2W-36
32.25/34.68

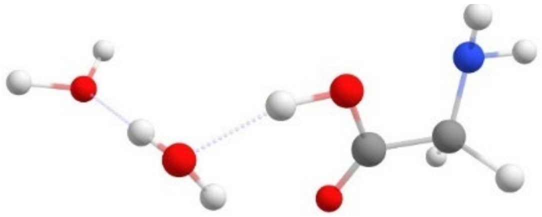

26-CIV-2W-62
32.70/25.25

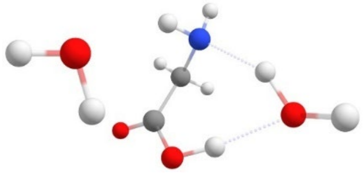

27-CIV-2W-48
33.97/34.85

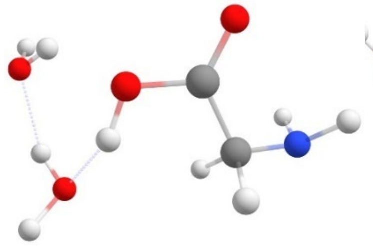

28-CIp-2W-56
36.83/32.51

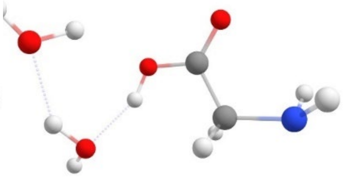

29-CIp-2W-38
37.23/32.89

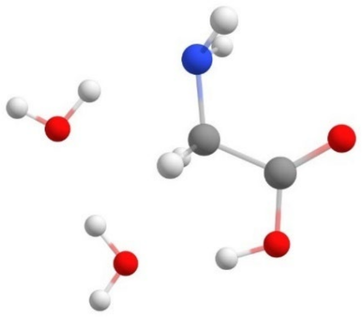

30-CIp-2W-32
38.46/34.81

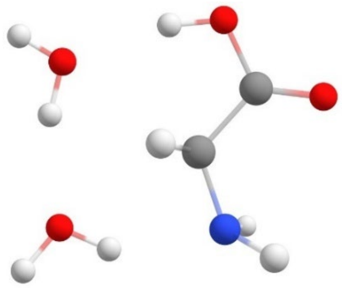

31-CIV-2W-37
38.71/36.51

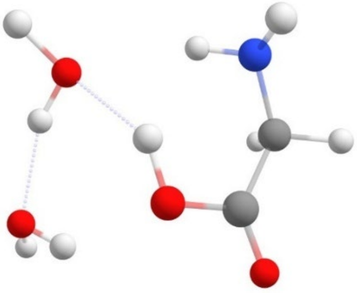

32-CIV-2W-50
39.38/37.88

Figure S5. Continuation. Most stable structures of canonical glycine with two water molecules in isolated conditions. Relative electronic energies considering the zero-point energy and relative Gibbs free energies at room temperature calculated using B3LYP-D3BJ/6-311++G(d,p) are also indicated ($\Delta E_{ZPE}/\Delta G$). Values are given in kJ/mol.

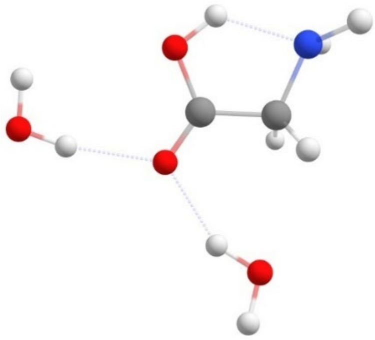

33-CIIp-2W-34
39.40/27.17

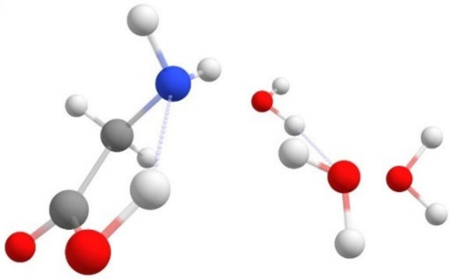

34-CIIp-2W-59
39.93/35.95

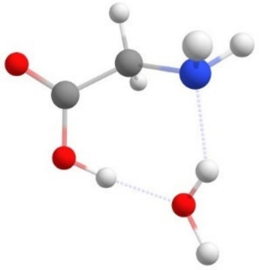

35-CIV-2W-51
42.03/38.16

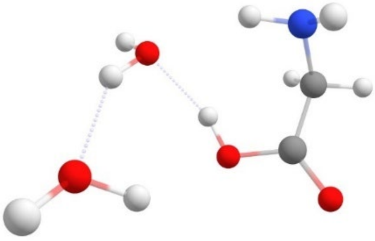

36-CIV-2W-60
42.96/41.74

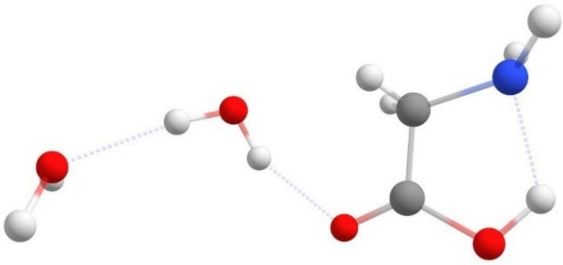

37-CIIp-2W-70
45.60/31.98

Table S3. Theoretical spectroscopic parameters for the calculated structures of Glycine-2H_2O at B3LYP-D3BJ/6-311++G(d,p).

Par.	1-CIp-2W-6	2-CIIIp-2W-66	3-CInp-2W-9	4-CIIIp-2W-7	5-CIIInp-2W-10	6-CIIp-2W-28	7-CIV-2W-19	8-CIIp-2W-15	9-CIIp-2W-4	10-CIIp-2W-12
A^a	3783	4158	3781	4135	4106	2313	2269	2407	2410	2414
B	1102	1064	1120	1064	1077	1734	1741	1763	1764	1762
C	864	858	875	857	869	1260	1217	1260	1262	1262
μ_a	-1.4	-1.2	-0.1	-1.3	0.3	0.4	-0.3	2.2	2.2	2.2
μ_b	0.5	-0.7	0.0	-0.6	-0.1	-1.9	-2.5	-2.3	-2.3	-2.3
μ_c	0.0	0.0	-1.3	2.2	-0.9	-1.0	2.2	0.0	0.0	0.0
χ_{aa}	-4.5079	-3.685	2.4475	-3.6939	2.4803	-1.1585	-1.0504	1.9511	1.9524	1.9549
χ_{aa}	2.4245	1.5803	2.9216	1.5898	2.663	2.1396	2.2684	0.9025	0.9122	0.9128
χ_{aa}	2.0833	2.1048	-5.3691	2.1042	-5.1433	-0.9811	-1.218	-2.8536	-2.8646	-2.8677
ΔE^b	0	4.07	5.26	6.48	11.47	15.82	16.21	16.85	16.85	16.85
ΔE_{ZPE}^c	0	4.25	4.99	5.87	10.89	16.45	16.47	17.27	17.29	17.31
ΔG^d	0	3.45	5.99	4.06	11.03	16.83	15.94	16.81	16.89	16.98

[a] *A*, *B* and *C* represent the rotation constants (in MHz); μ_a, μ_b and μ_c are the components of the electric dipole moment (in D). χ_{aa}, χ_{bb} y χ_{cc} are the diagonal elements of the ^{14}N nuclear quadrupole coupling tensor in MHz. [b]Relative energies (in kJ mol^{-1}) with respect to the global minimum. [c]Relative energies (in kJ mol^{-1}) with respect to the global minimum, considering the zero-point energy (ZPE).[d]Gibbs energies (in kJ mol^{-1}) calculated at 298 K and 1 atm.

Table S3. Continuation. Theoretical spectroscopic parameters for the calculated structures of Glycine-$2H_2O$ at B3LYP-D3BJ/6-311++G(d,p).

Par.	11-CIp-2W-30	12-CInp-2W-25	13-CIp-2W-41	14-CIV-2W-11	15-CInp-2W-16	16-CInp-2W-29	17-CIIIp-2W-31	18-CIp-2W-39	19-CInp-2W-61	20-CInp-2W-64
**A**[a]	7385	2413	2691	2558	5940	2998	7371	3616	2696	5138
**B**	739	1694	1336	1316	677	1169	744	1075	1340	716
**C**	678	1237	940	971	650	1012	683	940	929	687
μ_a	-2.3	-2.7	2.1	-0.6	4.6	-1.1	2.2	-1.2	-0.9	-2.7
μ_b	-2.4	-1.9	1.9	-2.2	0.3	1.4	2.6	-2.3	-0.4	1.3
μ_c	-1.0	1.5	-0.4	0.6	1.7	-1.6	0.9	0.1	0.9	-1.0
χ_{aa}	-4.2696	-2.5546	-0.6211	-0.5693	-3.703	2.0026	-4.4492	-1.3796	2.7247	2.2702
χ_{aa}	2.3856	2.861	-1.5773	0.785	2.1891	-4.024	2.5301	-0.8494	1.8416	1.5251
χ_{aa}	1.884	-0.3064	2.1983	-0.2157	1.5138	2.0214	1.9191	2.229	-4.5662	-3.7953
ΔE^b	21.86	17.56	23.91	23.35	25.82	24.46	27.16	29.25	28.75	31.48
$\Delta E_{ZPE}{}^c$	20.02	20.34	22.32	22.60	22.79	22.98	25.50	26.83	27.32	27.32
ΔG^d	13.49	23.39	20.99	19.50	14.59	18.32	18.15	22.46	26.59	17.61

[a] *A*, *B* and *C* represent the rotation constants (in MHz); μ_a, μ_b and μ_c are the components of the electric dipole moment (in D). χ_{aa}, χ_{bb} y χ_{cc} are the diagonal elements of the ^{14}N nuclear quadrupole coupling tensor in MHz. [b]Relative energies (in kJ mol^{-1}) with respect to the global minimum. [c]Relative energies (in kJ mol^{-1}) with respect to the global minimum, considering the zero-point energy (ZPE).[d]Gibbs energies (in kJ mol^{-1}) calculated at 298 K and 1 atm.

Table S3. Continuation. Theoretical spectroscopic parameters for the calculated structures of Glycine-2H2O at B3LYP-D3BJ/6-311++G(d,p).

Par.	21-CInp-2W-63	22-CIIIp-2W-21	23-CIV-2W-14	24-CIV-2W-35	25-CIV-2W-36	26-CIV-2W-62	27-CIV-2W-48	28-CIp-2W-56	29-CIp-2W-38	30-CIp-2W-32
A[a]	5523	5395	3427	2813	2946	4553	2214	3308	3183	2592
B	696	699	1282	1508	1301	746	1653	1082	1114	1453
C	671	659	995	1157	1160	699	1456	872	902	941
μ_a	-3.1	-4.4	5.1	0.8	-3.4	-2.2	-2.4	2.7	-3.6	5.8
μ_b	0.8	-2.3	-1.3	3.7	1.9	-2.3	-1.8	-1.6	-1.3	-0.8
μ_c	-1.1	1.1	0.3	0.3	1.9	0.0	2.6	-0.5	0.8	0.0
χ_{aa}	2.3783	-4.4583	-3.1413	-2.9493	1.8064	1.142	-1.4751	2.1494	2.2312	-4.4528
χ_{aa}	2.2227	2.9064	1.2272	2.7503	1.687	1.9985	2.1964	-4.3632	-4.4458	2.6078
χ_{aa}	-4.601	1.5519	1.914	0.199	-3.4934	-3.1405	-0.7212	2.2138	2.2146	1.845
ΔE[b]	30.94	30.70	27.32	27.62	30.21	35.65	33.85	39.01	39.78	39.21
ΔE_{ZPE}[c]	27.60	27.79	29.38	29.52	32.25	32.70	33.97	36.83	37.23	38.46
ΔG[d]	20.27	18.15	30.71	32.06	34.68	25.25	34.85	32.51	32.89	34.81

[a] *A*, *B* and *C* represent the rotation constants (in MHz); μ_a, μ_b and μ_c are the components of the electric dipole moment (in D). χ_{aa}, χ_{bb} y χ_{cc} are the diagonal elements of the ^{14}N nuclear quadrupole coupling tensor in MHz. [b]Relative energies (in kJ mol^{-1}) with respect to the global minimum. [c]Relative energies (in kJ mol^{-1}) with respect to the global minimum, considering the zero-point energy (ZPE).[d]Gibbs energies (in kJ mol^{-1}) calculated at 298 K and 1 atm.

Table S3. Continuation. Theoretical spectroscopic parameters for the calculated structures of Glycine-2H_2O at B3LYP-D3BJ/6-311++G(d,p).

Parameters	31-CIV-2W-37	32-CIV-2W-50	33-CIIp-2W-34	34-CIIp-2W-59	35-CIV-2W-51	36-CIV-2W-60	37-CIIp-2W-70
A^a	2598	2455	2067	3258	3114	2559	3916
B	1451	1495	1367	1073	1177	1424	739
C	941	990	833	927	914	1036	649
μ_a	5.8	-2.6	6.4	-4.2	-8.5	3.4	-0.4
μ_b	-0.9	-2.1	2.9	0.7	-0.8	-1.7	-1.5
μ_c	0.1	-0.1	-0.2	1.9	-1.2	-0.2	-0.5
χ_{aa}	-4.4519	-0.7551	-1.9122	1.7948	2.1184	0.2616	0.8349
χ_{aa}	2.6079	-0.1145	0.0302	-4.0256	-4.5428	-2.9877	-2.4971
χ_{aa}	1.844	0.8697	1.882	2.2308	2.4244	2.726	1.6622
ΔE^b	39.21	40.99	43.82	40.06	42.60	43.22	49.33
ΔE_{ZPE}^c	38.71	39.38	39.40	39.93	42.03	42.96	45.60
ΔG^d	36.51	37.88	27.17	35.95	38.16	41.74	31.98

[a] *A*, *B* and *C* represent the rotation constants (in MHz); μ_a, μ_b and μ_c are the components of the electric dipole moment (in D). χ_{aa}, χ_{bb} y χ_{cc} are the diagonal elements of the ^{14}N nuclear quadrupole coupling tensor in MHz. [b]Relative energies (in kJ mol^{-1}) with respect to the global minimum. [c]Relative energies (in kJ mol^{-1}) with respect to the global minimum, considering the zero-point energy (ZPE).[d]Gibbs energies (in kJ mol^{-1}) calculated at 298 K and 1 atm.

Figure S6. Most stable structures of zwitterionic glycine with two water molecules in isolated conditions. Relative electronic energies considering the zero-point energy and relative Gibbs free energies at room temperature calculated using B3LYP-D3BJ/6-311++G(d,p) are also indicated ($\Delta E_{ZPE}/\Delta G$). Values are given in kJ/mol.

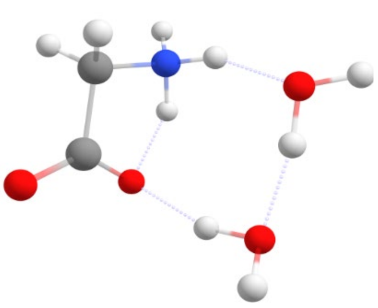

1-Z-2W-8
0/0

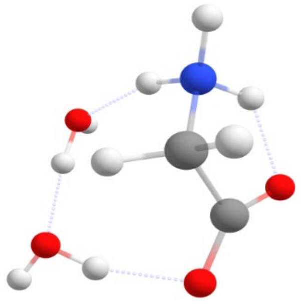

2-Z-2W-5
2.83/4.32

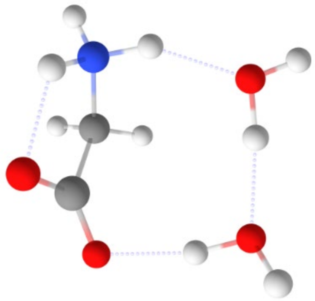

3-Z-2W-6
3.37/1.82

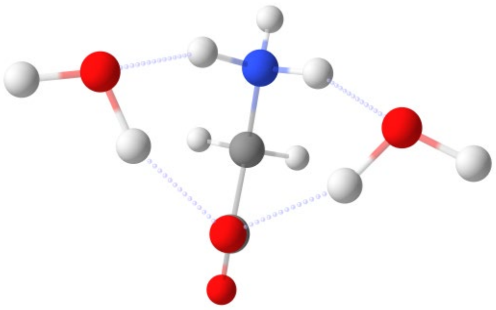

4-Z-2W-4
4.22/6.52

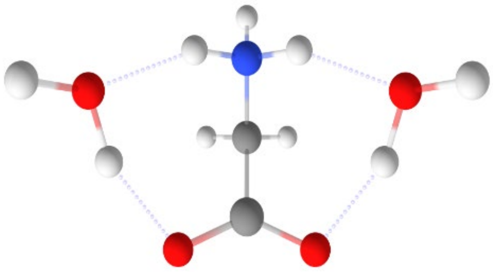

5-Z-2W-11
6.09/8.36

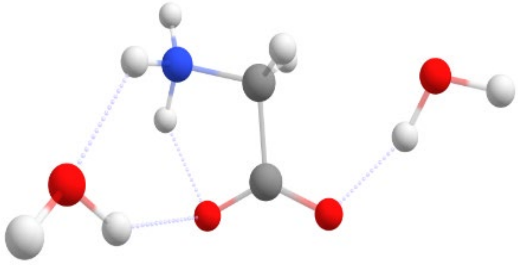

6-Z-2W-7
25.30/20.22

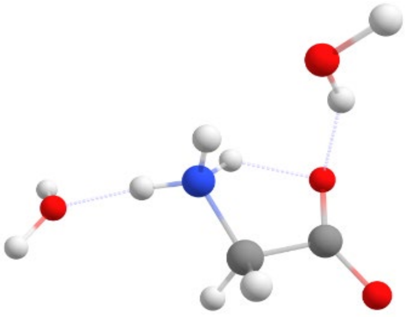

7-Z-2W-10
29.85/19.32

Table S4. Theoretical spectroscopic parameters for the calculated structures of zwitterionic Glycine and two water molecules at B3LYP-D3BJ/6-311++G(d,p).

Parameters	1-Z-2W-8	2-Z-2W-5	3-Z-2W-6	4-Z-2W-4	5-Z-2W-11	6-Z-2W-7	7-Z-2W-10
**A**[a]	2411	2306	2249	2206	2695	2939	2498
**B**	1720	1894	1929	1701	1667	1244	1153
**C**	1227	1283	1256	1471	1279	1139	880
μ_a	3.6	2.6	-2.0	4.2	0.0	3.4	10.1
μ_b	6.5	5.4	-6.9	0.0	6.9	5.4	-2.0
μ_c	-0.6	-2.1	-1.9	5.5	-0.7	0.5	1.7
χ_{aa}	-0.1111	-1.0839	-0.7705	0.6225	-1.226	0.1862	0.0884
χ_{aa}	0.4808	1.187	1.1389	-1.1248	1.2599	0.0041	0.5026
χ_{aa}	-0.3697	-0.1031	-0.3684	0.5022	-0.0339	-0.1904	-0.5909
ΔE^b	0	2.06	3.41	2.35	3.47	27.16	34.21
$\Delta E_{ZPE}{}^c$	0	2.83	3.37	4.22	6.09	25.30	29.85
ΔG^d	0	4.32	1.82	6.52	8.36	20.22	19.32

[a] *A*, *B* and *C* represent the rotation constants (in MHz); μ_a, μ_b and μ_c are the components of the electric dipole moment (in D). χ_{aa}, χ_{bb} y χ_{cc} are the diagonal elements of the ^{14}N nuclear quadrupole coupling tensor in MHz. [b]Relative energies (in kJ mol^{-1}) with respect to the global minimum. [c]Relative energies (in kJ mol^{-1}) with respect to the global minimum, considering the zero-point energy (ZPE). [d]Gibbs energies (in kJ mol^{-1}) calculated at 298 K and 1 atm.

Figure S7. Most stable structures of canonical glycine with one DMSO molecule in isolated conditions. Relative electronic energies considering the zero-point energy and relative Gibbs free energies at room temperature calculated using B3LYP-D3BJ/6-311++G(d,p) are also indicated ($\Delta E_{ZPE}/\Delta G$). Values are given in kJ/mol.

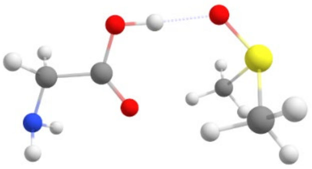

1-CIp-1DMSO-6
0/0

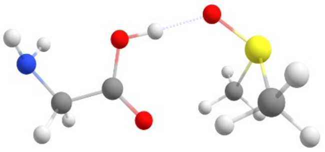

2-CIIIp-1DMSO-9
3.72/3.32

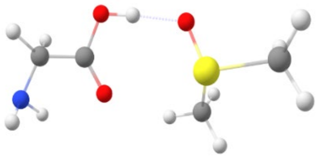

3-CIp-1DMSO-36
4.18/5.71

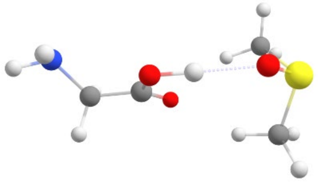

4-CIIInp-1DMSO-90
5.62/4.04

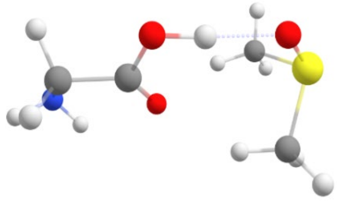

5-CInp-1DMSO-49
8.83/10.02

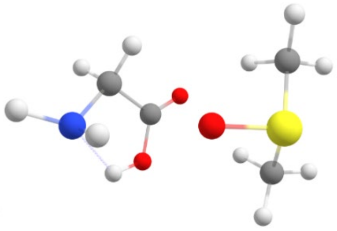

6-CIIp-1DMSO-25
16.14/18.31

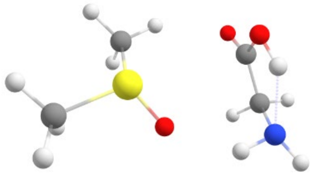

7-CIIp-1DMSO-4
21.11/20.47

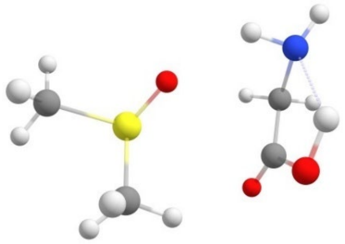

8-CIIp-1DMSO-67
22.07/20.64

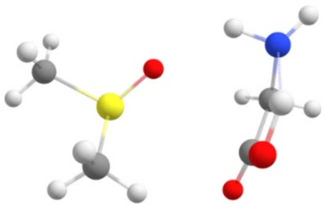

9-CIIp-1DMSO-19
22.16/19.20

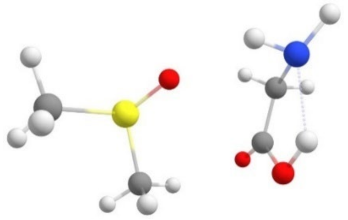

10-CIIp-1DMSO-11
22.35/18.47

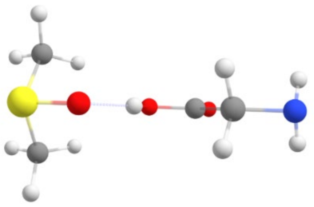

11-CIV-1DMSO-57
27.61/21.92

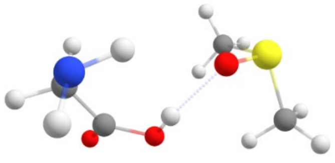

12-CIV-1DMSO-76
29.51/26.36

Figure S7. Continuation. Most stable structures of canonical glycine with one DMSO molecule in isolated conditions. Relative electronic energies considering the zero-point energy and relative Gibbs free energies at room temperature calculated using B3LYP-D3BJ/6-311++G(d,p) are also indicated ($\Delta E_{ZPE}/\Delta G$). Values are given in kJ/mol.

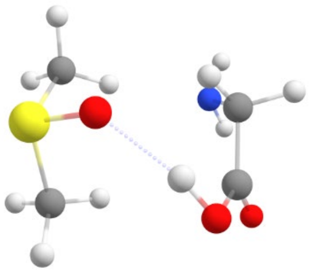

13-CInp-1DMSO-105
29.88/30.67

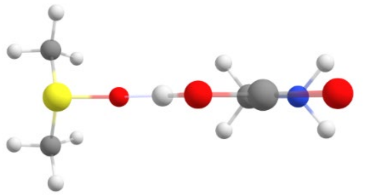

14-CIp-1DMSO-45
34.45/20.95

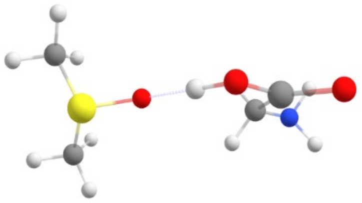

15-CIp-1DMSO-52
34.55/22.60

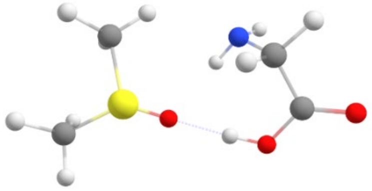

16-CIV-1DMSO-78
35.08/36.36

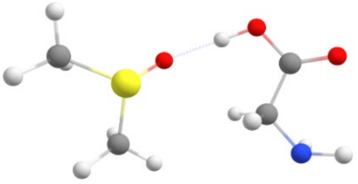

17-CIp-1DMSO-44
35.12/30.20

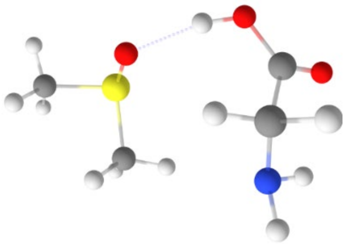

18-CInp-1DMSO-106
35.79/37.21

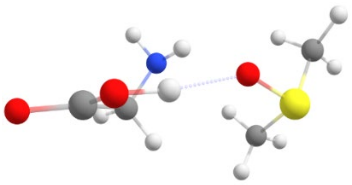

19-CIV-1DMSO-83
37.77/37.07

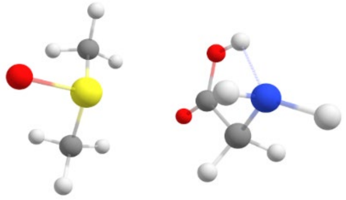

20-CIIp-1DMSO-94
40.16/38.88

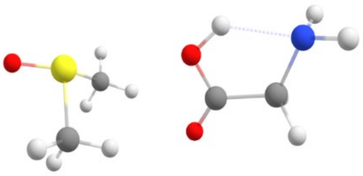

21-CIIp-1DMSO-104
50.01/33.60

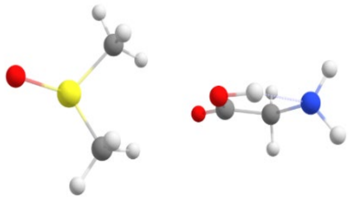

22-CIIp-1DMSO-100
51.70/38.62

Table S5. Theoretical spectroscopic parameters for the calculated structures of Glycine-DMSO at B3LYP-D3BJ/6-311++G(d,p).

Par.	1-CIp-1DMSO-6	2-CIIIp-1DMSO-9	3-CIp-1DMSO-36	4-CIIInp-1DMSO-90	5-CInp-1DMSO-49	6-CIIp-1DMSO-25	7-CIIp-1DMSO-4	8-CIIp-1DMSO-67	9-CIIp-1DMSO-19	10-CIIp-1DMSO-11
A[a]	2632	2836	2625	3334	2808	1869	2118	2130	2114	2081
B	588	573	598	543	580	870	788	782	778	776
C	549	544	557	492	553	792	662	668	667	665
μ_a	2.9	3.4	-1.7	-4.7	-1.9	-1.4	-3.4	3.1	3.0	-3.1
μ_b	3.3	2.0	-2.9	2.6	2.4	1.4	-2.2	2.3	-2.3	-2.3
μ_c	0.0	0.0	-1.2	-0.4	0.5	-0.6	0.1	0.3	0.3	0.3
χ_{aa}	-4.8327	-4.0373	2.3321	-4.9169	2.3966	0.9095	1.291	0.9880	1.0015	0.9891
χ_{aa}	2.7332	1.9442	2.9703	2.7961	2.7342	1.3691	2.4325	1.3474	1.3385	1.346
χ_{aa}	2.0995	2.0931	-5.3024	2.1208	-5.1309	-2.2786	-3.7235	-2.3355	-2.34	-2.3351
ΔE^b	0	3.53	4.32	6.27	8.84	13.76	19.50	20.65	20.73	20.86
$\Delta E_{ZPE}{}^c$	0	3.72	4.18	5.62	8.83	16.14	21.11	22.07	22.16	22.35
ΔG^d	0	3.32	5.71	4.04	10.02	18.31	20.47	20.64	19.20	18.47

[a] *A*, *B* and *C* represent the rotation constants (in MHz); μ_a, μ_b and μ_c are the components of the electric dipole moment (in D). χ_{aa}, χ_{bb} y χ_{cc} are the diagonal elements of the ^{14}N nuclear quadrupole coupling tensor in MHz. [b]Relative energies (in kJ mol^{-1}) with respect to the global minimum. [c]Relative energies (in kJ mol^{-1}) with respect to the global minimum, considering the zero-point energy (ZPE). [d]Gibbs energies (in kJ mol^{-1}) calculated at 298 K and 1 atm.

Table S5. Continuation. Theoretical spectroscopic parameters for the calculated structures of Glycine-DMSO at B3LYP-D3BJ/6-311++G(d,p).

Par.	11-ClV-1DMSO-57	12-ClV-1DMSO-76	13-Clnp-1DMSO-105	14-Clp-1DMSO-45	15-Clp-1DMSO-52	16-ClV-1DMSO-78	17-Clp-1DMSO-44	18-Clnp-1DMSO-106	19-ClV-1DMSO-83	20-Cllp-1DMSO-94
A[a]	2584	2020	1870	2615	2611	2674	2439	2310	2643	1801
B	526	698	829	451	459	626	573	682	601	830
C	493	614	723	428	425	546	482	583	562	750
μ_a	-6.2	-6.2	3.8	-9.7	-9.7	-9.1	-8.7	-6.2	-8.9	3.1
μ_b	-0.7	-0.3	4.0	2.2	2.2	2.6	3.4	4.3	3.2	1.1
μ_c	0.0	0.8	0.9	0.0	0.0	-0.9	-0.6	1.1	0.5	1.5
χ_{aa}	2.9623	0.543	-3.9033	2.4687	2.4687	-0.1205	0.8409	-3.3938	0.084	1.361
χ_{aa}	-5.0098	-1.5394	2.7441	-4.5127	-4.5127	-1.8437	-2.767	2.9264	-2.5958	1.9639
χ_{aa}	2.0474	0.9965	1.1591	2.044	2.044	1.9641	1.926	0.4674	2.5118	-3.325
ΔE[b]	28.65	29.15	28.10	35.92	36.01	32.90	35.68	34.03	35.59	39.44
ΔE_{ZPE}[c]	27.61	29.51	29.88	34.45	34.55	35.08	35.12	35.79	37.77	40.16
ΔG[d]	21.92	26.36	30.67	20.95	22.60	36.36	30.20	37.21	37.07	38.88

[a] *A*, *B* and *C* represent the rotation constants (in MHz); μ_a, μ_b and μ_c are the components of the electric dipole moment (in D). χ_{aa}, χ_{bb} y χ_{cc} are the diagonal elements of the ^{14}N nuclear quadrupole coupling tensor in MHz. [b]Relative energies (in kJ mol^{-1}) with respect to the global minimum. [c]Relative energies (in kJ mol^{-1}) with respect to the global minimum, considering the zero-point energy (ZPE).[d]Gibbs energies (in kJ mol^{-1}) calculated at 298 K and 1 atm.

Table S5. Continuation. Theoretical spectroscopic parameters for the calculated structures of Glycine-DMSO at B3LYP-D3BJ/6-311++G(d,p).

Parameters	21-ClIp-1DMSO-104	22-ClIp-1DMSO-100
A[a]	3014	3456
B	472	428
C	456	422
μ_a	10.1	-10.6
μ_b	-0.2	0.2
μ_c	0.0	-0.1
χ_{aa}	-2.649	-1.7448
χ_{aa}	0.759	-0.1276
χ_{aa}	1.8901	1.8725
ΔE[b]	51.82	53.44
ΔE_{ZPE}[c]	50.02	51.70
ΔG[d]	33.56	38.62

[a] *A*, *B* and *C* represent the rotation constants (in MHz); μ_a, μ_b and μ_c are the components of the electric dipole moment (in D). χ_{aa}, χ_{bb} y χ_{cc} are the diagonal elements of the ^{14}N nuclear quadrupole coupling tensor in MHz. [b]Relative energies (in kJ mol^{-1}) with respect to the global minimum. [c]Relative energies (in kJ mol^{-1}) with respect to the global minimum, considering the zero-point energy (ZPE).[d]Gibbs energies (in kJ mol^{-1}) calculated at 298 K and 1 atm.

Figure S8. Most stable structures of canonical glycine with two DMSO molecules in isolated conditions. Relative electronic energies considering the zero-point energy and relative Gibbs free energies at room temperature calculated using B3LYP-D3BJ/6-311++G(d,p) are also indicated ($\Delta E_{ZPE}/\Delta G$). Values are given in kJ/mol.

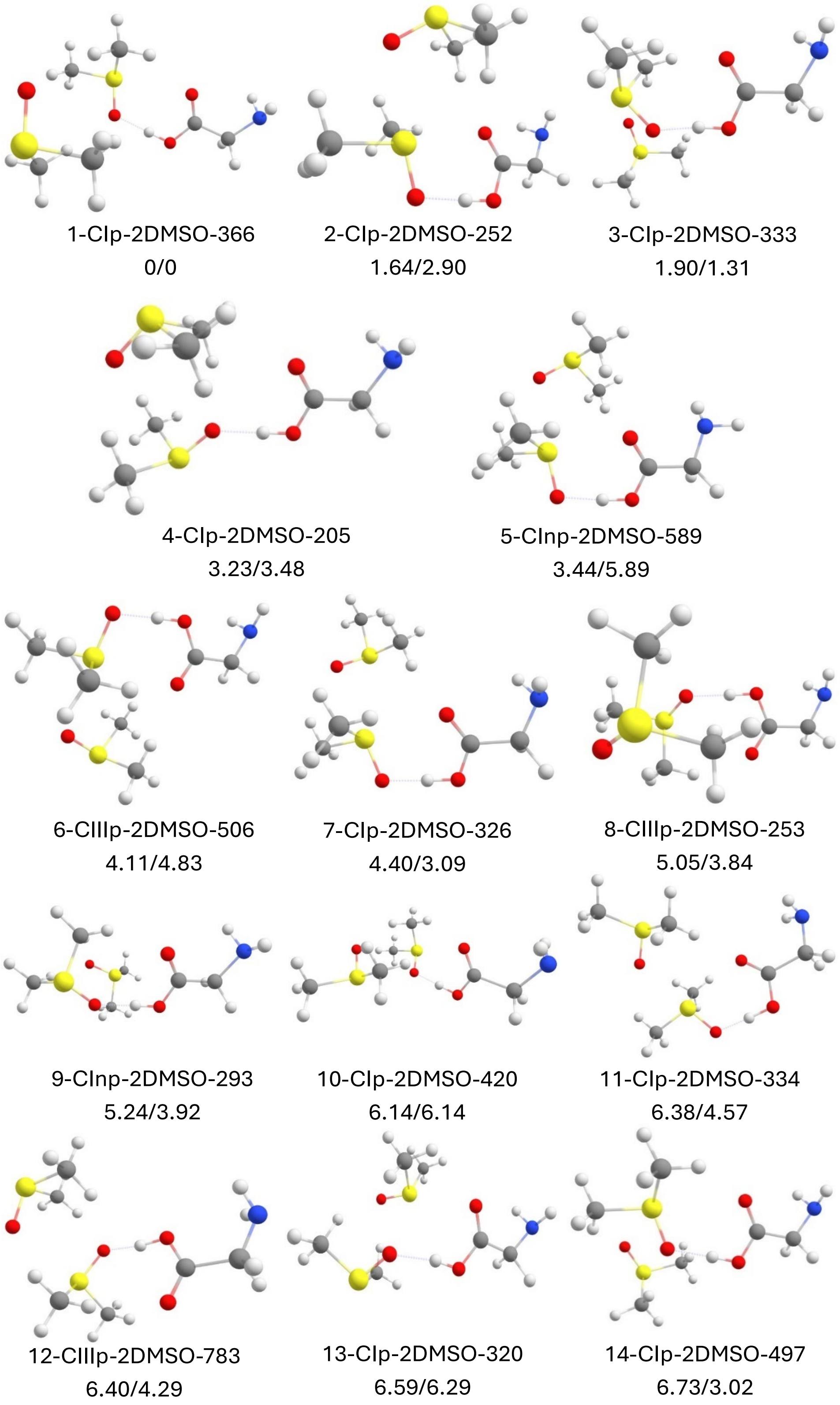

Figure S8. Continuation. Most stable structures of canonical glycine with two DMSO molecules in isolated conditions. Relative electronic energies considering the zero-point energy and relative Gibbs free energies at room temperature calculated using B3LYP-D3BJ/6-311++G(d,p) are also indicated ($\Delta E_{ZPE}/\Delta G$). Values are given in kJ/mol.

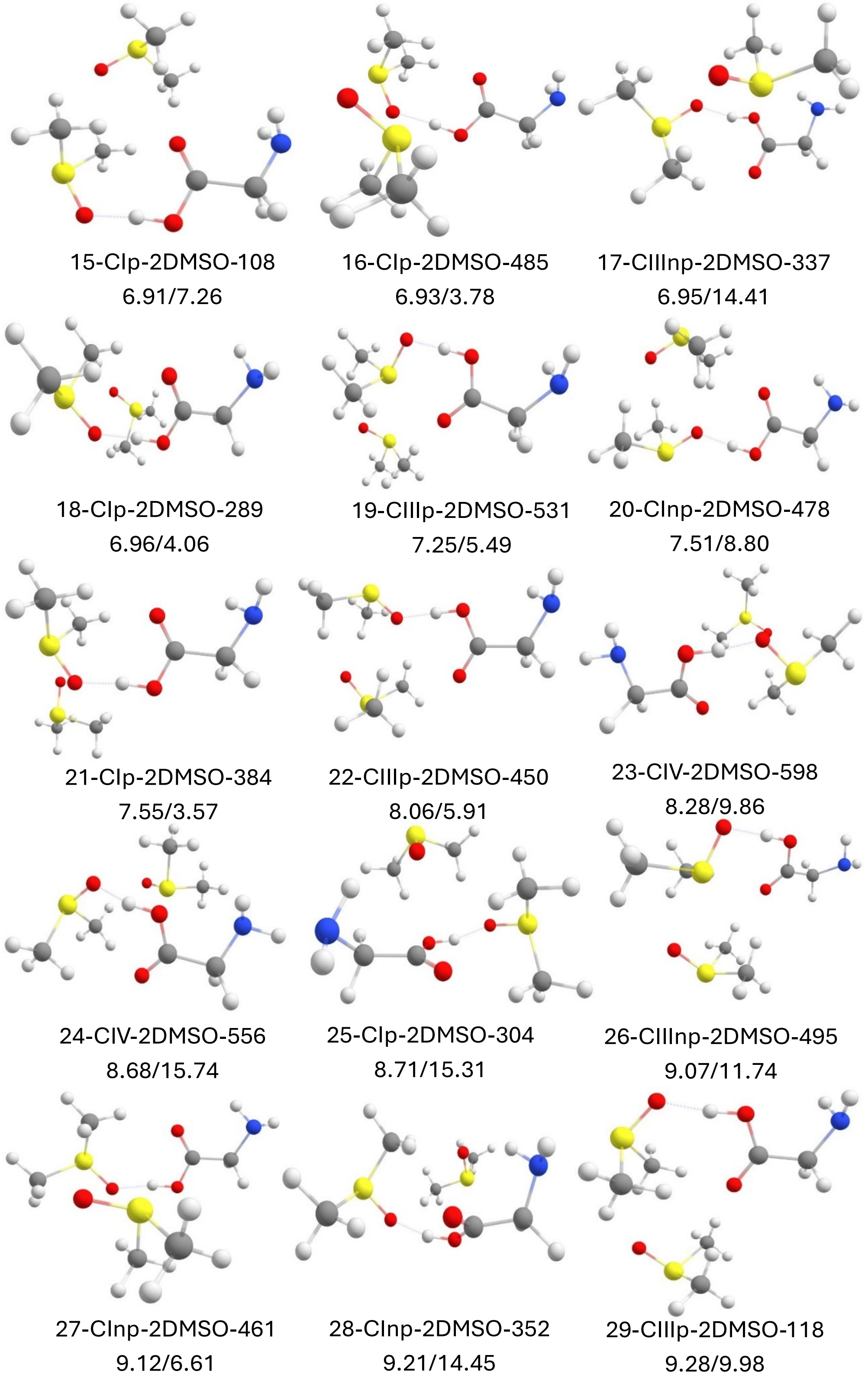

Figure S8. Continuation. Most stable structures of canonical glycine with two DMSO molecules in isolated conditions. Relative electronic energies considering the zero-point energy and relative Gibbs free energies at room temperature calculated using B3LYP-D3BJ/6-311++G(d,p) are also indicated ($\Delta E_{ZPE}/\Delta G$). Values are given in kJ/mol.

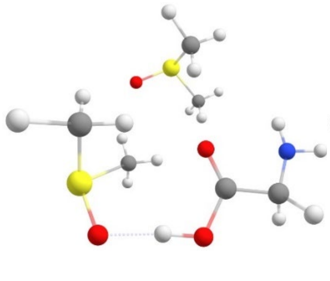

30-CInp-2DMSO-559
9.36/11.52

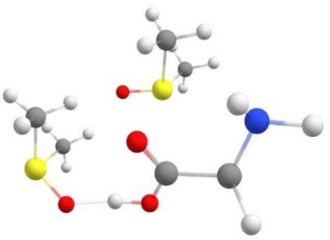

31-CInp-2DMSO-345
9.39/12.37

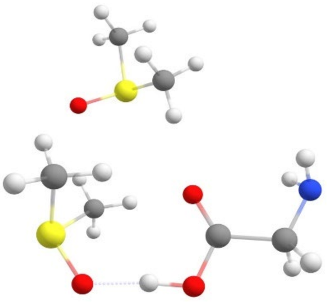

32-CIp-2DMSO-112
9.40/7.75

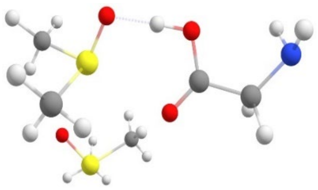

33-CIIIp-2DMSO-383
9.55/7.43

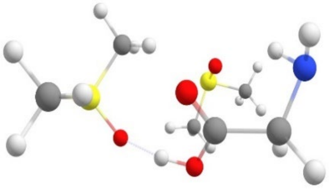

34-CIp-2DMSO-72
10.15/14.11

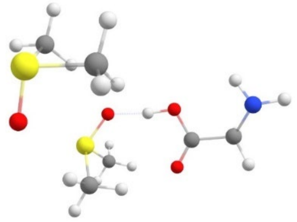

35-CIIInp-2DMSO-603
10.23/10.43

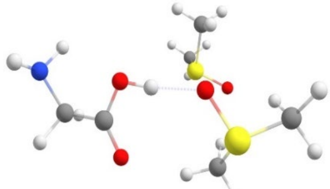

36-CIIInp-2DMSO-373
10.42/6.85

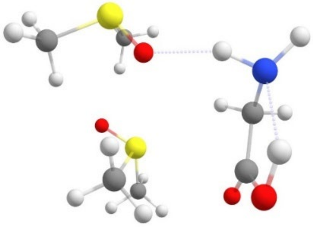

37-CIIp-2DMSO-595
10.57/14.99

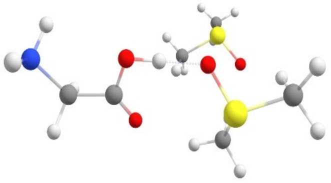

38-CIIInp-2DMSO-511
10.61/10.19

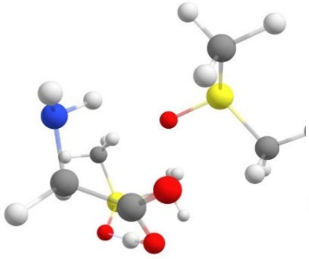

39-CIV-2DMSO-138
10.63/17.07

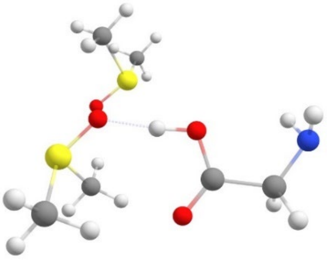

40-CIIIp-2DMSO-557
11.07/6.25

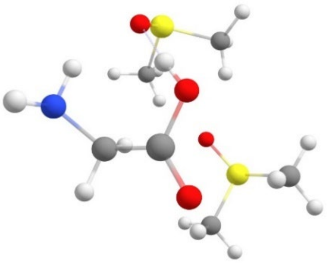

41-CIV-2DMSO-517
11.13/16.50

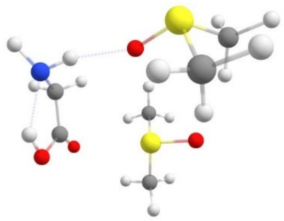

42-CIIp-2DMSO-236
11.27/16.15

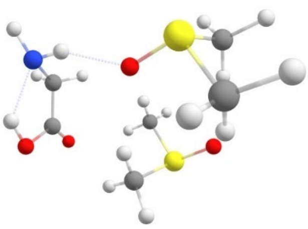

43-CIIp-2DMSO-521
11.46/17.90

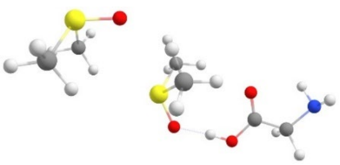

44-CIp-2DMSO-388
11.60/7.56

Figure S8. Continuation. Most stable structures of canonical glycine with two DMSO molecules in isolated conditions. Relative electronic energies considering the zero-point energy and relative Gibbs free energies at room temperature calculated using B3LYP-D3BJ/6-311++G(d,p) are also indicated ($\Delta E_{ZPE}/\Delta G$). Values are given in kJ/mol.

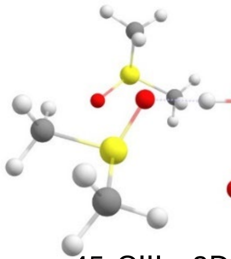

45-CIIIp-2DMSO-600
11.64/6.23

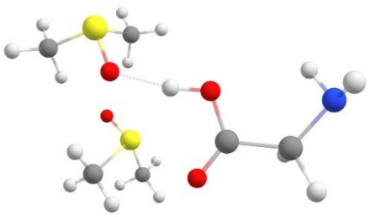

46-CIIIp-2DMSO-412
11.64/10.42

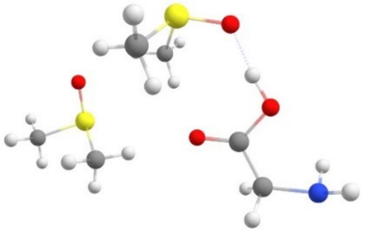

47-CIIIp-2DMSO-135
11.85/9.56

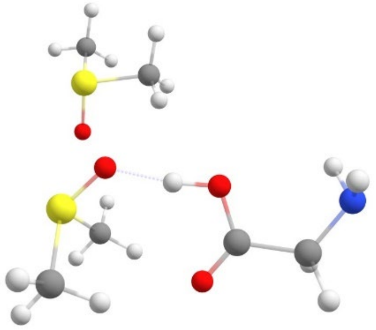

48-CIIIp-2DMSO-472
11.99/6.09

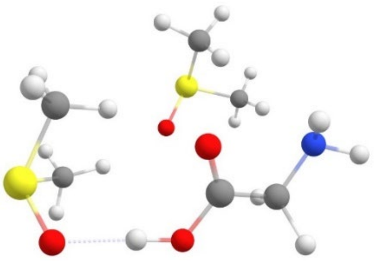

49-CIV-2DMSO-281
11.99/15.53

Table S6. Theoretical spectroscopic parameters for the calculated structures of Glycine-2DMSO at B3LYP-D3BJ/6-311++G(d,p).

Par.	1-CIp-2DMSO-366	2-CIp-2DMSO-252	3-CIp-2DMSO-333	4-CIp-2DMSO-205	5-CInp-2DMSO-589	6-CIIIp-2DMSO-506	7-CIp-2DMSO-326	8-CIIIp-2DMSO-253	9-CInp-2DMSO-293
A[a]	801	620	796	698	607	698	654	714	604
B	284	401	286	318	429	348	336	319	384
C	245	277	240	246	288	262	239	262	279
μ_a	-2.2	2.5	1.0	-2.5	-0.5	2.5	-4.9	-1.8	-2.6
μ_b	0.4	-3.0	0.8	1.4	-3.3	2.2	-3.2	-0.4	-1.1
μ_c	0.6	0.3	1.0	0.0	1.1	-0.2	0.4	1.3	0.0
χ_{aa}	-2.7617	-4.0091	-0.9608	-4.5878	0.8486	0.1686	-3.1487	-3.3919	-2.1071
χ_{aa}	0.1367	1.8404	-1.45	2.4889	2.1651	-2.2874	0.9636	0.9509	-0.4849
χ_{aa}	2.625	2.1687	2.4108	2.0989	-3.0137	2.1189	2.185	2.4411	2.592
ΔE[b]	0	3.60	2.83	3.41	5.55	6.07	6.48	4.79	6.17
ΔE_{ZPE}[c]	0	1.64	1.90	3.23	3.44	4.11	4.40	5.05	5.24
ΔG[d]	0	2.90	1.31	3.48	5.89	4.83	3.09	3.84	3.92

[a] *A*, *B* and *C* represent the rotation constants (in MHz); μ_a, μ_b and μ_c are the components of the electric dipole moment (in D). χ_{aa}, χ_{bb} y χ_{cc} are the diagonal elements of the ^{14}N nuclear quadrupole coupling tensor in MHz. [b]Relative energies (in kJ mol^{-1}) with respect to the global minimum. [c]Relative energies (in kJ mol^{-1}) with respect to the global minimum, considering the zero-point energy (ZPE). [d]Gibbs energies (in kJ mol^{-1}) calculated at 298 K and 1 atm.

Table S6. Continuation. Theoretical spectroscopic parameters for the calculated structures of Glycine-2DMSO at B3LYP-D3BJ/6-311++G(d,p).

Par.	10-CIp-2DMSO-420	11-CIp-2DMSO-334	12-CIIIp-2DMSO-783	13-CIp-2DMSO-320	14-CIp-2DMSO-497	15-CIp-2DMSO-108	16-CIp-2DMSO-485	17-CIIInp-2DMSO-337	18-CIp-2DMSO-289	19-CIIIp-2DMSO-531
A[a]	692	646	707	706	706	520	521	713	519	750
B	324	334	313	358	317	427	435	440	437	296
C	251	240	247	272	243	266	281	333	282	228
μ_a	2.6	5.2	-1.2	-2.6	-1.2	-3.4	0.2	1.4	0.2	4.8
μ_b	0.2	2.9	0.1	0.3	1.2	-2.7	0.5	-1.1	0.5	2.5
μ_c	-0.7	0.5	-1.5	0.5	-0.9	0.0	0.3	-0.6	-0.3	-0.2
χ_{aa}	-3.1901	-3.0519	-3.8621	-1.9657	-1.1789	0.6324	1.8151	1.0552	1.8716	0.5136
χ_{aa}	0.6525	0.8178	1.9123	1.3625	-1.3349	-2.7864	-4.4191	-2.7603	-4.4785	-2.7019
χ_{aa}	2.5376	2.2341	1.9498	0.6032	2.5137	2.154	2.604	1.7051	2.6069	2.1884
ΔE[b]	7.19	8.72	7.23	7.70	8.53	8.75	8.29	6.42	8.29	9.22
ΔE_{ZPE}[c]	6.14	6.38	6.40	6.59	6.73	6.91	6.93	6.95	6.96	7.25
ΔG[d]	6.14	4.57	4.29	6.29	3.02	7.26	3.78	14.41	4.06	5.49

[a] *A*, *B* and *C* represent the rotation constants (in MHz); μ_a, μ_b and μ_c are the components of the electric dipole moment (in D). χ_{aa}, χ_{bb} y χ_{cc} are the diagonal elements of the ^{14}N nuclear quadrupole coupling tensor in MHz. [b]Relative energies (in kJ mol^{-1}) with respect to the global minimum. [c]Relative energies (in kJ mol^{-1}) with respect to the global minimum, considering the zero-point energy (ZPE).[d]Gibbs energies (in kJ mol^{-1}) calculated at 298 K and 1 atm.

Table S6. Continuation. Theoretical spectroscopic parameters for the calculated structures of Glycine-2DMSO at B3LYP-D3BJ/6-311++G(d,p).

Par.	20-CInp-2DMSO-478	21-CIp-2DMSO-384	22-CIIIp-2DMSO-450	23-CIV-2DMSO-598	24-CIV-2DMSO-556	25-CIp-2DMSO-304	26-CIIInp-2DMSO-495	27-CInp-2DMSO-461	28-CInp-2DMSO-352	29-CIIIp-2DMSO-118
A[a]	697	809	697	665	679	680	678	611	682	586
B	323	272	317	359	444	427	361	402	416	359
C	248	224	245	277	324	321	270	293	311	251
μ_a	-1.3	-2.5	-2.0	0.3	0.6	-2.2	-1.1	-0.7	-0.3	-2.5
μ_b	-1.8	0.4	-2.5	0.3	-3.1	-3.3	-2.9	-0.8	4.1	-2.9
μ_c	1.1	-0.3	0.0	-1.3	0.0	0.1	1.2	0.6	-0.8	0.0
χ_{aa}	2.3957	-0.3365	-4.3217	-0.2412	2.546	-3.6714	1.4881	2.1426	-2.9278	2.3272
χ_{aa}	2.8908	-2.0623	2.2116	-0.9172	-4.5237	0.3748	1.0174	-4.0533	-0.4323	-4.4036
χ_{aa}	-5.2866	2.3988	2.1101	1.1584	1.9777	3.2965	-2.5055	1.9107	3.3601	2.0764
ΔE[b]	7.94	9.92	8.22	7.84	8.51	7.08	10.98	10.24	7.92	10.90
ΔE_{ZPE}[c]	7.51	7.55	8.06	8.28	8.68	8.71	9.07	9.12	9.21	9.28
ΔG[d]	8.80	3.57	5.91	9.86	15.74	15.31	11.74	9.61	14.45	9.98

[a] *A*, *B* and *C* represent the rotation constants (in MHz); μ_a, μ_b and μ_c are the components of the electric dipole moment (in D). χ_{aa}, χ_{bb} y χ_{cc} are the diagonal elements of the ^{14}N nuclear quadrupole coupling tensor in MHz. [b]Relative energies (in kJ mol^{-1}) with respect to the global minimum. [c]Relative energies (in kJ mol^{-1}) with respect to the global minimum, considering the zero-point energy (ZPE).[d]Gibbs energies (in kJ mol^{-1}) calculated at 298 K and 1 atm.

Table S6. Continuation. Theoretical spectroscopic parameters for the calculated structures of Glycine-2DMSO at B3LYP-D3BJ/6-311++G(d,p).

Par.	30-CInp-2DMSO-559	31-CInp-2DMSO-345	32-CIp-2DMSO-112	33-CIIIp-2DMSO-383	34-CIp-2DMSO-72	35-CIIInp-2DMSO-603	36-CIIInp-2DMSO-373	37-CIIp-2DMSO-595	38-CIIInp-2DMSO-511	39-CIV-2DMSO-138
A[a]	524	688	571	741	692	662	597	677	688	712
B	440	394	342	297	410	343	391	417	327	401
C	272	298	230	229	315	258	280	305	252	340
μ_a	2.7	3.3	-5.6	-4.9	-0.5	1.0	-2.6	-3.6	-2.6	-0.9
μ_b	3.7	2.8	-3.1	-2.2	3.7	0.1	-1.5	-2.1	-0.2	5.6
μ_c	1.0	-1.2	0.4	0.6	-0.5	2.0	-0.9	-1.0	-0.2	1.2
χ_{aa}	1.9076	2.3028	-0.0505	0.7343	-3.3936	0.015	-3.1828	-0.5024	-3.304	0.8243
χ_{aa}	2.5016	-4.6529	-2.0702	-2.8318	0.2222	2.7022	1.0257	2.7133	0.7956	0.8379
χ_{aa}	-4.4092	2.3501	2.1206	2.0975	3.1714	-2.7172	2.1571	-2.211	2.5084	-1.6622
ΔE[b]	11.32	10.85	11.03	11.76	9.56	11.31	11.38	8.48	11.47	8.17
ΔE_{ZPE}[c]	9.36	9.39	9.40	9.55	10.15	10.23	10.42	10.57	10.61	10.63
ΔG[d]	11.52	12.37	7.75	7.43	14.11	10.43	6.85	14.99	10.19	17.07

[a] *A*, *B* and *C* represent the rotation constants (in MHz); μ_a, μ_b and μ_c are the components of the electric dipole moment (in D). χ_{aa}, χ_{bb} y χ_{cc} are the diagonal elements of the ^{14}N nuclear quadrupole coupling tensor in MHz. [b]Relative energies (in kJ mol^{-1}) with respect to the global minimum. [c]Relative energies (in kJ mol^{-1}) with respect to the global minimum, considering the zero-point energy (ZPE).[d]Gibbs energies (in kJ mol^{-1}) calculated at 298 K and 1 atm.

Table S6. Continuation. Theoretical spectroscopic parameters for the calculated structures of Glycine-2DMSO at B3LYP-D3BJ/6-311++G(d,p).

Par.	40-CIIIp-2DMSO-557	41-CIV-2DMSO-517	42-CIIp-2DMSO-236	43-CIIp-2DMSO-521	44-CIp-2DMSO-388	45-CIIIp-2DMSO-600	46-CIIIp-2DMSO-412	47-CIIIp-2DMSO-135	48-CIIIp-2DMSO-472	49-CIV-2DMSO-281
A[a]	584	681	708	630	1516	657	639	655	714	708
B	360	406	411	413	188	341	383	294	297	390
C	256	318	312	300	182	250	278	217	229	301
μ_a	-2.0	-1.2	-3.5	-2.2	7.6	-1.2	-2.6	-4.9	-2.8	3.7
μ_b	0.1	5.3	-2.0	-4.5	-0.4	0.6	-0.9	-3.1	0.3	3.8
μ_c	1.1	-1.2	1.1	1.2	0.0	1.5	-0.7	-0.4	-0.8	-1.0
χ_{aa}	-2.3315	2.733	0.9031	0.1916	-5.1961	-3.4928	-4.207	2.3437	-3.3762	1.8571
χ_{aa}	0.4155	2.1199	2.3416	2.8813	3.1057	1.5238	2.9777	-4.4198	1.5134	-4.0293
χ_{aa}	1.9161	-4.8529	-3.2447	-3.0729	2.0904	1.969	1.2293	2.0761	1.8628	2.1722
ΔE[b]	12.49	8.89	8.90	7.94	13.38	13.32	12.45	13.47	14.30	12.85
ΔE_{ZPE}[c]	11.07	11.13	11.27	11.46	11.60	11.64	11.64	11.85	11.99	11.99
ΔG[d]	6.25	16.50	16.15	17.90	7.56	6.23	10.42	9.56	6.09	15.53

[a] *A*, *B* and *C* represent the rotation constants (in MHz); μ_a, μ_b and μ_c are the components of the electric dipole moment (in D). χ_{aa}, χ_{bb} y χ_{cc} are the diagonal elements of the ^{14}N nuclear quadrupole coupling tensor in MHz. [b]Relative energies (in kJ mol^{-1}) with respect to the global minimum. [c]Relative energies (in kJ mol^{-1}) with respect to the global minimum, considering the zero-point energy (ZPE). [d]Gibbs energies (in kJ mol^{-1}) calculated at 298 K and 1 atm.

Figure S9. Most stable structures of zwitterionic glycine with one DMSO molecule in isolated conditions. Relative electronic energies considering the zero-point energy and relative Gibbs free energies at room temperature calculated using B3LYP-D3BJ/6-311++G(d,p) are also indicated ($\Delta E_{ZPE}/\Delta G$). Values are given in kJ/mol.

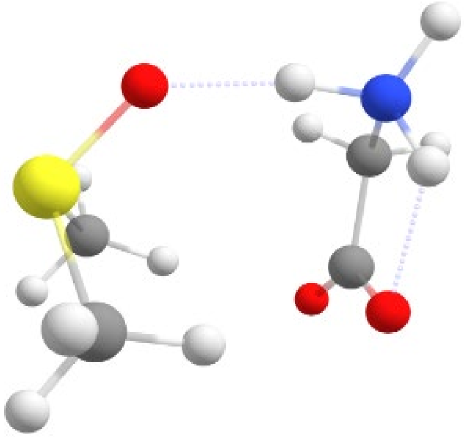

1-Z-1DMSO-2
0/0

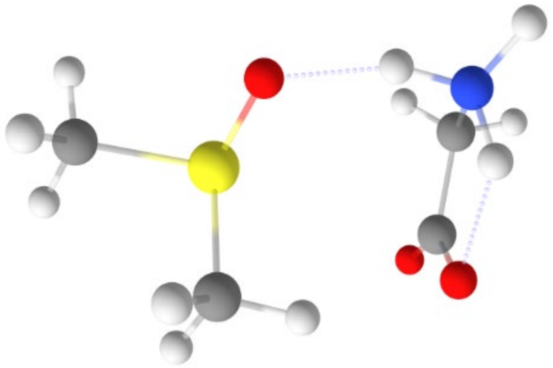

2-Z-1DMSO-3
10.97/7.53

Table S7. Theoretical spectroscopic parameters for the calculated structures of zwitterionic glycine and one DMSO molecule at B3LYP-D3BJ/6-311++G(d,p).

Parameters	1-Z-1DMSO-2	2-Z-1DMSO-3
A^a	1775	2090
B	897	786
C	798	691
μ_a	-2.6	4.6
μ_b	4.4	-5.0
μ_c	-0.8	0.5
χ_{aa}	-0.9876	-0.6819
χ_{aa}	1.301	0.8945
χ_{aa}	-0.3134	-0.2126
ΔE^b	0	12.75
$\Delta E_{ZPE}{}^c$	0	10.97
ΔG^d	0	7.53

[a] *A*, *B* and *C* represent the rotation constants (in MHz); μ_a, μ_b and μ_c are the components of the electric dipole moment (in D). χ_{aa}, χ_{bb} y χ_{cc} are the diagonal elements of the ^{14}N nuclear quadrupole coupling tensor in MHz. [b]Relative energies (in kJ mol^{-1}) with respect to the global minimum. [c]Relative energies (in kJ mol^{-1}) with respect to the global minimum, considering the zero-point energy (ZPE). [d]Gibbs energies (in kJ mol^{-1}) calculated at 298 K and 1 atm.

Figure S10. Most stable structures of zwitterionic glycine with two DMSO molecules in isolated conditions. Relative electronic energies considering the zero-point energy and relative Gibbs free energies at room temperature calculated using B3LYP-D3BJ/6-311++G(d,p) are also indicated ($\Delta E_{ZPE}/\Delta G$). Values are given in kJ/mol.

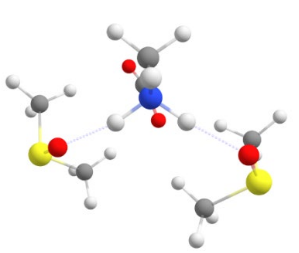

1-Z-2DMSO-7
0/0

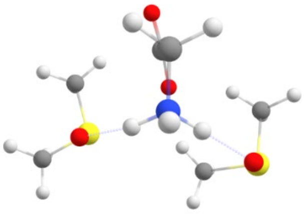

2-Z-2DMSO-33
4.83/5.57

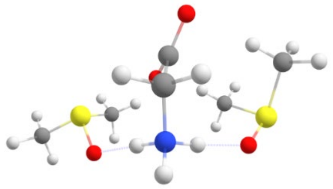

3-Z-2DMSO-34
8.77/6.38

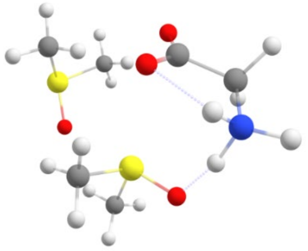

4-Z-2DMSO-40
11.36/12.14

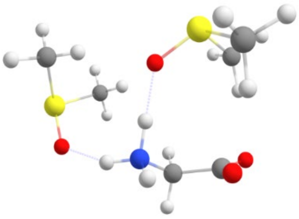

5-Z-2DMSO-23
11.78/10.90

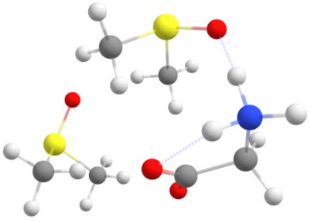

6-Z-2DMSO-43
12.25/14.82

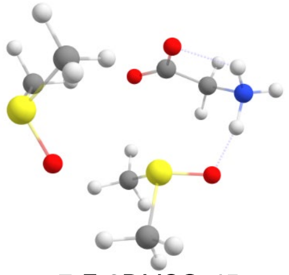

7-Z-2DMSO-45
12.34/15.56

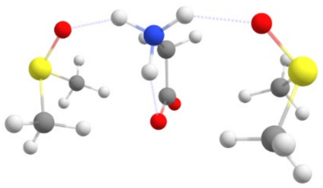

8-Z-2DMSO-1
12.62/3.03

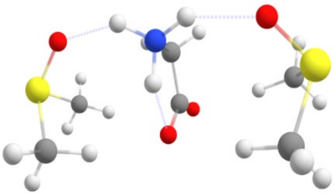

9-Z-2DMSO-4
12.83/7.29

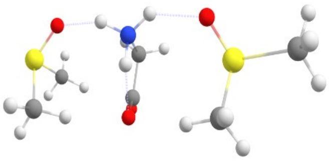

10-Z-2DMSO-25
17.93/13.38

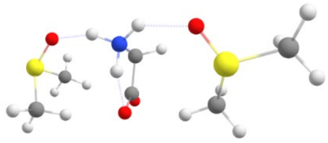

11-Z-2DMSO-9
18.03/13.45

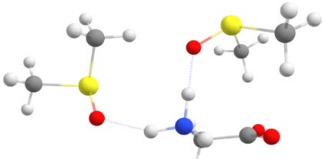

12-Z-2DMSO-19
18.37/13.29

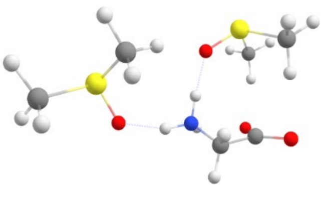

13-Z-2DMSO-31
18.55/12.99

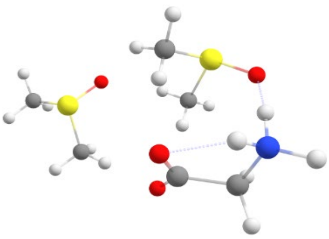

14-Z-2DMSO-41
23.34/20.56

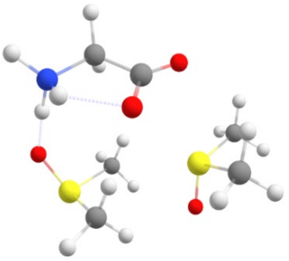

15-Z-2DMSO-39
24.02/24.34

Figure S10. Continuation. Most stable structures of zwitterionic glycine with two DMSO molecules in isolated conditions. Relative electronic energies considering the zero-point energy and relative Gibbs free energies at room temperature calculated using B3LYP-D3BJ/6-311++G(d,p) are also indicated ($\Delta E_{ZPE}/\Delta G$). Values are given in kJ/mol.

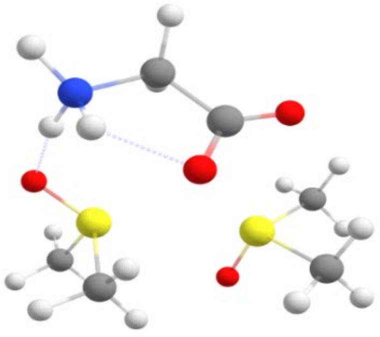

16-Z-2DMSO-44
25.58/23.24

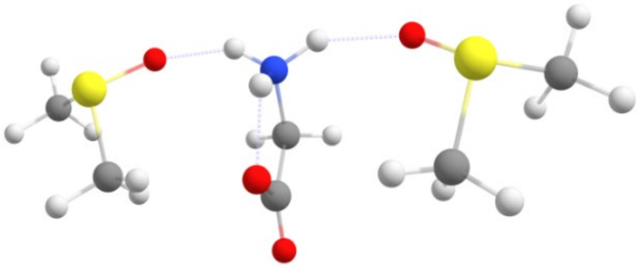

17-Z-2DMSO-05
26.14/15.28

Table S8. Theoretical spectroscopic parameters for the calculated structures of zwitterionic glycine and two DMSO molecules at B3LYP-D3BJ/6-311++G(d,p).

Parameters	1-Z-2DMSO-7	2-Z-2DMSO-33	3-Z-2DMSO-34	4-Z-2DMSO-40	5-Z-2DMSO-23	6-Z-2DMSO-43	7-Z-2DMSO-45	8-Z-2DMSO-1	9-Z-2DMSO-4
**A**[a]	836	729	928	685	725	706	689	1094	1077
**B**	319	360	288	450	344	390	449	257	258
**C**	292	305	272	327	279	304	335	245	244
μ_a	-0.7	0.7	2.5	-3.0	-5.7	3.1	-2.8	0.0	-0.6
μ_b	3.1	5.2	3.1	1.2	-6.7	1.3	1.3	-1.6	-1.7
μ_c	0.5	0.9	1.6	-0.2	0.3	-1.1	1.6	-1.6	-1.7
χ_{aa}	-1.3669	-1.4529	-1.4479	1.1425	-0.254	1.0742	0.8312	-0.2894	-0.3387
χ_{aa}	0.2404	0.2682	0.0995	-1.1593	-0.5735	-1.2853	-1.3771	0.773	0.7933
χ_{aa}	1.1265	1.1847	1.3484	0.0168	0.8275	0.211	0.5459	-0.4836	-0.4545
**ΔE**[b]	0	5.52	9.98	14.90	11.94	14.29	14.82	14.67	14.63
ΔE_{ZPE}[c]	0	4.83	8.77	11.36	11.78	12.25	12.34	12.62	12.83
**ΔG**[d]	0	5.57	6.38	12.14	10.90	14.82	15.56	3.03	7.29

[a] *A*, *B* and *C* represent the rotation constants (in MHz); μ_a, μ_b and μ_c are the components of the electric dipole moment (in D). χ_{aa}, χ_{bb} y χ_{cc} are the diagonal elements of the ^{14}N nuclear quadrupole coupling tensor in MHz. [b]Relative energies (in kJ mol^{-1}) with respect to the global minimum. [c]Relative energies (in kJ mol^{-1}) with respect to the global minimum, considering the zero-point energy (ZPE). [d]Gibbs energies (in kJ mol^{-1}) calculated at 298 K and 1 atm.

Table S8. Continuation. Theoretical spectroscopic parameters for the calculated structures of zwitterionic glycine and two DMSO molecules at B3LYP-D3BJ/6-311++G(d,p).

Parameters	10-Z-2DMSO-25	11-Z-2DMSO-9	12-Z-2DMSO-19	13-Z-2DMSO-31	14-Z-2DMSO-41	15-Z-2DMSO-39	16-Z-2DMSO-44	17-Z-2DMSO-05
A[a]	1179	1177	778	779	762	650	606	1206
B	230	229	291	286	340	462	525	214
C	219	215	238	240	269	328	338	195
μ_a	-2.8	3.0	-8.2	-7.6	-1.0	-3.9	-4.2	0.0
μ_b	2.1	-2.0	-6.4	-6.3	-0.1	-1.5	-1.2	-3.1
μ_c	-1.7	-1.0	0.1	-0.7	-0.4	0.8	1.0	-0.4
χ_{aa}	-0.4234	-0.3929	-0.1114	-0.1956	1.113	0.8001	0.493	-0.297
χ_{aa}	0.4201	0.5006	-0.6399	-0.6106	-1.2269	-0.5629	-0.0296	0.4429
χ_{aa}	0.0033	-0.1077	0.7513	0.8062	0.1139	-0.2372	-0.4633	-0.1459
ΔE[b]	19.93	19.75	19.45	19.60	26.48	27.11	29.67	29.44
ΔE_{ZPE}[c]	17.93	18.03	18.37	18.55	23.34	24.02	25.58	26.14
ΔG[d]	13.38	13.45	13.29	12.99	20.56	24.34	23.24	15.28

[a] *A*, *B* and *C* represent the rotation constants (in MHz); μ_a, μ_b and μ_c are the components of the electric dipole moment (in D). χ_{aa}, χ_{bb} y χ_{cc} are the diagonal elements of the ^{14}N nuclear quadrupole coupling tensor in MHz. [b]Relative energies (in kJ mol^{-1}) with respect to the global minimum. [c]Relative energies (in kJ mol^{-1}) with respect to the global minimum, considering the zero-point energy (ZPE). [d]Gibbs energies (in kJ mol^{-1}) calculated at 298 K and 1 atm.

Table S9. Topological properties at the bond critical points (BCP) for the most stable glycine conformer calculated using the QTAIM analysis at B3LYP/6-311++G(d,p).

Glycine				
Bond	ρ^a	$\nabla^2\rho^b$	V/G	H^e
C_1-O_2	0.419	-0.154	2.057	-0.714
C_1-C_5	0.253	-0.610	4.519	-0.213
C_1-O_3	0.296	-0.484	2.400	-0.424
O_3-H_4	0.358	-2.504	11.361	-0.693
C_5-H_6	0.278	-0.937	8.029	-0.273
C_5-H_7	0.278	-0.937	8.030	-0.273
C_5-N_9	0.274	-0.733	3.699	-0.291
H_8-N_9	0.338	-1.511	8.300	-0.438
N_9-H_{10}	0.338	-1.511	8.300	-0.438

[a] Electron density at the BCP. [b] Laplacian at the BCP (au). [c] Potential energy density (au). [d] Gradient kinetic energy density (au). [e] Total electronic energy density (au). [f] Bond ellipticity.

Table S10. Topological properties at the bond critical points (BCP) for the most stable conformer of Glycine with one and two water molecules and the zwitterionic form of glycine with one water molecule calculated using the QTAIM analysis at B3LYP/6-311++G(d,p). Only the relevant parameters are shown.

	Glycine-H_2O / Glycine-$2H_2O$ / GlycineZ-$2H_2O$				
	Bond	ρ^a	$\nabla^2\rho^b$	V/G	H^e
Gly-H_2O	H_{10}-O_{11}	0.036	0.120	1.021	-0.001
	O_5-H_{12}	0.025	0.088	0.918	0.002
Gly-$2H_2O$	H_7-O_{11}	0.050	0.139	1.154	-0.006
	H_{12}-O_{14}	0.040	0.132	1.048	-0.002
	O_5-H_{16}	0.034	0.121	0.974	0.001
Glyz-$2H_2O$	H_4-O_9	0.055	0.147	1.166	-0.007
	O_9-H_{13}	0.039	0.129	1.047	-0.002
	O_{11}-H_{16}	0.043	0.132	1.092	-0.003
	H_{10}-O_{14}	0.040	0.132	1.044	-0.002

[a] Electron density at the BCP. [b] Laplacian at the BCP (au). [c] Potential energy density (au). [d] Gradient kinetic energy density (au). [e] Total electronic energy density (au). [f] Bond

Table S11. Topological properties at the bond critical points (BCP) for the most stable conformer of Glycine and its zwitterionic form with one DMSO molecule calculated using the QTAIM analysis at B3LYP/6-311++G(d,p). Only the relevant parameters are shown.

	Glycine-DMSO / GlycineZ-DMSO				
	Bond	ρ^a	$\nabla^2\rho^b$	V/G	H^e
Gly-DMSO	H_7-O_{12}	0.0559	0.1528	1.199	-0.009
	O_2-H_{14}	0.0130	0.0431	0.839	0.001
	O_2-H_{20}	0.0130	0.0431	0.839	0.001
Glyz-DMSO	O_6-H_{10}	0.043	0.137	1.034	-0.001
	H_7-O_{12}	0.057	0.155	1.199	-0.010
	O_2-H_{16}	0.014	0.044	0.832	0.002
	O_6-H_{19}	0.017	0.057	0.849	0.002

[a] Electron density at the BCP. [b] Laplacian at the BCP (au). [c] Potential energy density (au). [d] Gradient kinetic energy density (au). [e] Total electronic energy density (au). [f] Bond

Table S12. Topological properties at the bond critical points (BCP) for the most stable conformer of Glycine and its zwitterionic form with two DMSO molecules calculated using the QTAIM analysis at B3LYP/6-311++G(d,p). Only the relevant parameters are shown.

	Glycine-2DMSO / GlycineZ-2DMSO				
	Bond	ρ^a	$\nabla^2\rho^b$	V/G	H^e
Gly-2DMSO	H_{16}-O_{22}	0.014	0.045	0.875	0.001
	H_{20}-O_{22}	0.015	0.051	0.861	0.002
	O_{12}-H_{29}	0.017	0.056	0.860	0.002
	H_7-O_{22}	0.057	0.155	1.206	-0.010
	O_2-H_{26}	0.011	0.037	0.830	0.001
	O_{12}-H_{25}	0.016	0.053	0.867	0.002
Glyz-2DMSO	H_7-O_{12}	0.051	0.146	1.155	-0.007
	O_2-H_{14}	0.018	0.063	0.850	0.002
	O_2-H_{19}	0.017	0.065	0.812	0.003
	H_9-O_{22}	0.048	0.147	1.124	-0.005
	O_2-H_{24}	0.019	0.065	0.860	0.002
	O_6-H_{30}	0.015	0.048	0.835	0.002

[a] Electron density at the BCP. [b] Laplacian at the BCP (au). [c] Potential energy density (au). [d] Gradient kinetic energy density (au). [e] Total electronic energy density (au). [f] Bond
