# Supplementary material for: Stabilization of Zwitterionic Versus Canonical Glycine by DMSO Molecules
Source: Pharmaceuticals (Basel). 2025 Aug 6;18(8):1168. doi: 10.3390/ph18081168 (PMC12389192; doi:10.3390/ph18081168)
Supplement: Supplementary file 1 [file pharmaceuticals-18-01168-s001.zip › XYZ/pharmaceuticals-SI-xyz.pdf]

Supporting information for:

Stabilization of Zwitterionic versus Canonical Glycine by DMSO Molecules

We include the whole list of .xyz coordinates obtained from Molecular Mechanics (MM) after optimizing the structures using B3LYP-GD3BJ/6-311++G(d,p) in the xyz folder. For each system there is also an Excel file with all parameters and sorted by energy. Note that some of the structures converge into the same structure.

Finally, because the xyz labelling is that of MM, all the files in the Manuscript can be easily found as it matches with the last number of each structure. For example, structure 2-CIIIp-1W-4 corresponds to "gly_water4.xyz" inside the Gly-W1 folder.
